# Broadly protective bispecific antibodies that simultaneously target influenza virus hemagglutinin and neuraminidase

**DOI:** 10.1128/mbio.01085-24

**Published:** 2024-06-20

**Authors:** Kevin E. Ramos, Nisreen M. A. Okba, Jessica Tan, Pooja Bandawane, Philip S. Meade, Madhumathi Loganathan, Benjamin Francis, Sergey Shulenin, Frederick W. Holtsberg, M. Javad Aman, Meagan McMahon, Florian Krammer, Jonathan R. Lai

**Affiliations:** 1 Department of Biochemistry, Albert Einstein College of Medicine, Bronx, New York, USA; 2 Department of Microbiology, Icahn School of Medicine at Mount Sinai, New York, New York, USA; 3 Center for Vaccine Research and Pandemic Preparedness (C-VaRPP), Icahn School of Medicine at Mount Sinai, New York, New York, USA; 4 Integrated BioTherapeutics, Inc., Rockville, Maryland, USA; 5 Department of Pathology, Molecular and Cell-Based Medicine, Icahn School of Medicine at Mount Sinai, New York, New York, USA; 6 Ignaz Semmelweis Institute, Interuniversity Institute for Infection Research, Medical University of Vienna, Vienna, Austria; Dartmouth College, Hanover, New Hampshire, USA

**Keywords:** influenza, hemagglutinin, neuraminidase, monoclonal antibody, bispecific antibody, antibody engineering, immunotherapy

## Abstract

Monoclonal antibodies (mAbs) are an attractive therapeutic platform for the prevention and treatment of influenza virus infection. There are two major glycoproteins on the influenza virion surface: hemagglutinin (HA), which is responsible for viral attachment and entry, and neuraminidase (NA), which mediates viral egress by enzymatically cleaving sialic acid to release budding particles from the host cell surface. Broadly neutralizing antibodies (bNAbs) that target the conserved HA central stalk region, such as CR9114, can inhibit both viral entry and egress. More recently, broadly binding mAbs that engage and inhibit the NA active site, such as 1G01, have been described to prevent viral egress. Here, we engineered bispecific antibodies (bsAbs) that combine the variable domains of CR9114 and 1G01 into a single molecule and evaluated if simultaneous targeting of two different glycoproteins improved antiviral properties *in vitro* and *in vivo*. Several CR9114/1G01 bsAbs were generated with various configurations of the two sets of the variable domains (“bsAb formats”). We found that combinations employing the addition of a single-chain variable fragment in the hinge region of an IgG scaffold had the best properties in terms of expression, stability, and binding. Further characterization of selected bsAbs showed potent neutralizing and egress-inhibiting activity. One such bsAb (“hSC_CR9114_1G01”) provided higher levels of prophylactic protection from mortality and morbidity upon challenge with H1N1 than either of the parental mAbs at low dosing (1 mg/kg). These results highlight the potential use of bsAbs that simultaneously target HA and NA as new influenza immunotherapeutics.

**IMPORTANCE:**

Infection by the influenza virus remains a global health burden. The approaches utilized here to augment the activity of broadly protective influenza virus antibodies may lead to a new class of immunotherapies with enhanced activity.

## INTRODUCTION

Influenza viruses belong to the *Orthomyxoviridae* family of negative-sense RNA viruses. The main influenza viruses of concern that infect humans are influenza A virus (IAV) and influenza B virus (IBV), which cause about ~4 million severe infections worldwide and lead to 290,000 to 650,000 deaths annually. The surface of the membrane-bound influenza virion is decorated with two major and essential glycoproteins: hemagglutinin (HA) and neuraminidase (NA). HA is a trimeric class I (α-helical) fusion protein that is responsible for cell attachment by binding to sialic acid through the hypervariable head domain and viral membrane fusion via the highly conserved stalk domain. NA is a tetrameric enzyme that mediates the cleavage of sialic acid on N-linked glycans on host cell membranes and the resulting release of budding virions (1). Within IAV, there are 19 HA and 11 NA subtypes that are combined in various assortments to define host specificity, transmissibility, and pathogenicity (2). Reassortment of HA and NA (antigenic shift) within hosts can result in pandemic influenza virus strains such as the 1918 H1N1, 1957 H2N2, 1968 H3N2, and 2009 H1N1 pandemic influenza viruses (3). For IBV, there are two antigenically distinct lineages, B/Victoria/2/1987-like and B/Yamagata/16/1988-like, which harbor diverse HA and NA sequences. Further genetic variation within IAV subtypes or IBV lineages exists, which can lead to viral escape from past immunity (antigenic drift) or resistance to antiviral therapies over time (4). Thus, a critical objective for influenza virus vaccines and therapeutics is the development of agents that have broad efficacy against multiple subtypes and variants.

The characterization of protective human monoclonal antibodies (mAbs) from influenza virus-infected patients and vaccinated individuals has defined key sites of broad susceptibility on both HA and NA. For HA, broadly neutralizing antibodies (bNAbs), such as CR9114 and CR6261, target the conserved stalk domain, while others target the sialic acid-binding site on the HA head domain (5–8). NA-specific inhibitory antibodies (Abs), such as 1G01, target the conserved NA active site (5–7). These Abs are strong immunotherapeutic candidates, but the structural studies of Ab Fab-antigen complexes also reveal key sites of susceptibility that can be exploited for epitope-focused vaccine design. The development of mAbs and vaccines has historically focused on HA, but recently, the presence of NA Abs has been correlated with protection in humans. Furthermore, NA is critical at several stages of the viral lifecycle and thus represents a second attractive target for mAb or vaccine development (9–11).

MAbs are a promising therapeutic platform for viral infections because they are highly specific with few off-target effects, have a long serum half-life due to the Fc region, and are well-tolerated (12). However, mAb therapies targeting a single epitope are susceptible to viral escape by a single mutation and may have limited breadth, especially against viruses with high genetic diversity. bNAbs discovered for influenza viruses, ebolaviruses, alphaviruses, flaviviruses, and many others typically target highly conserved regions of the envelope glycoprotein, reducing the possibility of viral escape (13–21). Additionally, broadly protective antibodies that target conserved epitopes and mediate protection by non-neutralizing mechanisms have also been described for several viruses (20, 22).

Potentially, the risk of viral escape could be further mitigated by combining two or more broad antibodies as either a cocktail or a bispecific antibody (bsAb). bsAbs are engineered by fusing the variable domains (Fvs) of two different specificities into a single molecule, thus allowing bsAbs to engage two different epitopes. bsAb therapeutics are advantageous because they can bind to multiple epitopes (within a single antigen or on different antigens) simultaneously. In some cases, bsAbs have been engineered such that the physical linkage of the two Fvs confers synergistic effects (23). For example, we have previously reported “Trojan horse” bsAbs for filoviruses in which one “arm” is directed toward viral or host epitopes that shuttle the bsAb into endosomal compartments, where the other “arm” binds to the conserved receptor-binding domain on the viral glycoprotein that is only exposed after degradation by host endosomal proteases (24, 25). Another example is our development of the Crimean Congo hemorrhagic fever virus (CCHFV) bsAbs that outperformed cocktails of their parents in a therapeutic model (26). Additionally, a single bsAb is easier to manufacture than a cocktail of two different mAbs (27).

In this study, we explored whether simultaneous targeting of HA and NA in a bsAb conferred advantages for antiviral activity. HA is present on the viral surface in higher abundance than NA; thus, there is potential for enhanced anti-NA activity by a proximity effect of direct physical linkage between the two specificities. Furthermore, any reductions in potency that result from partial ablation of binding affinity due to viral mutation of either antigen could potentially be overcome in an HA/NA bsAb, again by high abundance on the viral surface. We incorporated the Fvs of CR9114 (HA-specific) and 1G01 (NA-specific) into several different bsAb scaffolds. For the constructs that expressed well and remained stable, we examined their binding. We then selected the best bsAbs to characterize their neutralizing, anti-NA activity, and protective capabilities. We found that some bsAbs displayed high binding reactivity and affinities toward different types of HA and NA antigens, neutralized viral mutants that were not susceptible to one of the parental monospecific mAbs, and were more potent at protecting mice from challenge with a suite of influenza viruses. This work highlights the effectiveness of targeting different influenza viral life-cycle stages by simultaneously targeting HA and NA and can offer insights about the development of new broad influenza therapeutics.

## RESULTS

### Design, expression, and purification of influenza bsAbs

The large sequence diversity of HA and NA across different influenza virus strains represents a significant challenge for the development of therapeutic antibodies. Although bNAbs against HA exist, the most potent of these, the stalk-binding Abs, target a compact epitope that is susceptible to changes that allow viral escape. In order to overcome the limitations of targeting a single epitope on a single antigen and to potentially realize antiviral synergy by targeting two different stages of the viral life cycle, we engineered bsAbs that can bind highly conserved epitopes on both HA and NA. We selected CR9114 (HA stalk binder) and 1G01 (NA active site binder) as the parental antibodies because both antibodies exhibit broad protective activity *in vivo* (28–30). Additionally, both mAbs inhibit NA either directly by binding to the NA active site (1G01) or indirectly by steric hindrance upon binding to the HA stalk (CR9114). Finally, such bsAbs may mitigate viral escape by targeting, via different mechanisms, various stages in the viral lifecycle: CR9114 inhibits viral entry and egress by a steric mechanism, and 1G01 prevents viral egress by blocking the NA active site.

**Fig 1:**
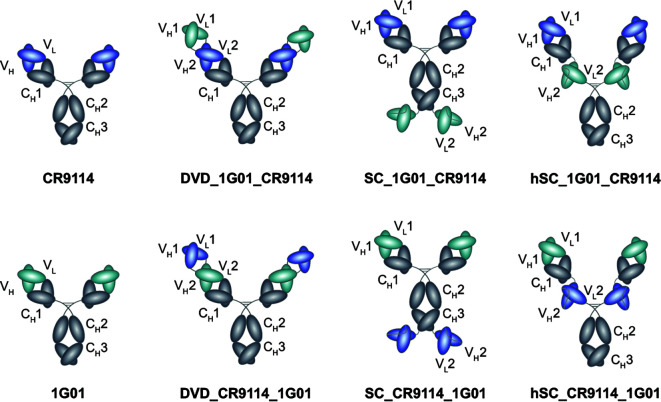
bsAb designs. Fvs of parental mAbs CR9114 and 1G01 colored blue and cyan, respectively. Three bsAb formats were explored: dual variable domain-Ig; scFv-Ig fusions (“SC”); and hinge scFv-Ig (“hSC”). For each format, constructs were created with the parental Fvs in both possible orientations.

We engineered three types of bsAb formats (Fig. 1). First, we developed the dual-variable-domain (DVD)-Ig bsAb, which has been shown to be highly stable and to exhibit IgG-like physicochemical and pharmacokinetic properties (31, 32). We have previously used the DVD-Ig format to successfully engineer antiviral bsAbs against filoviruses and bunyaviruses. For example, we reported broadly neutralizing ebolavirus “Trojan Horse” DVD-Ig bsAbs that combined the variable domains of a host-directed Niemann-Pick C1 (NPC1) mAb and a broadly reactive but non-neutralizing filovirus glycoprotein (GP) mAb. Ebolaviruses utilize NPC1 as a receptor to trigger viral membrane fusion in the endosome, but NPC1 is only available for binding deep in the endocytic pathway and cannot be accessed at the cell surface by conventional mAbs. Thus, the dual targeting of both the virus and the receptor allowed for the bsAbs to be internalized along with the virus into endosomal compartments where the second set of Fvs could block receptor binding (24). More recently, we reported bsAbs against CCHFV that fused the Fvs from non-competing mAbs targeting the Gc subunit into the DVD-Ig bsAb format and were observed to enhance neutralization and therapeutic protection *in vivo* (26). Here, influenza virus-specific DVD-Igs were constructed by genetically fusing the variable domains of one mAb to the variable domains of another using short polypeptide linkers as was done previously for “Trojan Horse” and CCHFV DVD-Igs.

In the second bsAb format, the single chain Fv (scFv) of one mAb was fused to the C-terminus of a second IgG backbone (scFv-Ig, abbreviated “SC”). We have previously reported scFv-Ig bsAbs that combined Fvs of an Ebola virus (Zaire)-specific antibody with a Sudan virus-specific antibody to produce a cross-protective bsAb (23, 33, 34). Finally, we also engineered “hinge scFv” (hSC) bsAbs, which contain a full-length IgG1 Ab backbone with single chain Fvs inserted into the hinge region. The hSCs also have IgG1-like biochemical and biophysical properties and are amenable to Fc engineering to extend half-life and modulate effector functions (35, 36).

We designed two configurations for each bsAb format, where we swapped the Fv positions of CR9114 and 1G01 to explore any potential differences in binding and inhibitory properties that may be associated with Fv orientation in any of the formats. DNA encoding each of the bsAbs as well as parental mAbs was cloned into the pMAZ-IgH and pMAZ-IgL dual plasmid system and purified from ExpiCHO cells as previously described (21, 24). Although we were successful in isolating most of the bsAbs designed, DVD_CR9114_1G01 expressed poorly and thus could not be isolated. Furthermore, both scFv-Ig (“SC”) designs (SC_CR9114_1G01 and SC_1G01_CR9114) were unstable due to proteolytic degradation as visualized by sodium dodecyl-sulfate polyacrylamide gel electrophoresis (SDS-PAGE) and thus were not further characterized. The other three bsAbs (DVD_1G01_CR9114, hSC_1G01_CR9114, and hSC_CR9114_1G01) as well as the parental mAbs exhibited the expected banding pattern in SDS-PAGE under both reducing and non-reducing conditions (Fig. S1). Yields for mAbs and bsAbs ranged from 5 to 25 mg/100 mL culture.

To further assess the stability of the antibodies, we assessed their susceptibility to degradation or aggregation over time. We used size-exclusion chromatography (SEC) to analyze samples of hSC_1G01_CR9114 and hSC_CR9114_1G01 as well as parental mAbs stored at 4°C for up to 1.5 years after purification (Fig. S2). The major species for all mAbs and bsAbs was the properly assembled (LC_2_HC_2_) “monomer” (>82%); bsAb hSC_CR9114_1G01 showed a small shoulder to the monomer peak (aggregates), but neither of the parental mAbs nor hSC_1G01_CR9114 contained this aggregate (Fig. S2D). We found minimal differences in the SEC profiles of all samples stored at 4°C for 1 day, 7 days, or 1.5 years, which indicates that bsAbs are stable for long-term storage. Additionally, we analyzed samples that were subjected to two freeze-thaw cycles (first frozen to −80°C then thawed and frozen again at −20°C and then thawed) and again observed no differences in SEC profiles (Fig. S2).

### Binding studies

**Fig 2:**
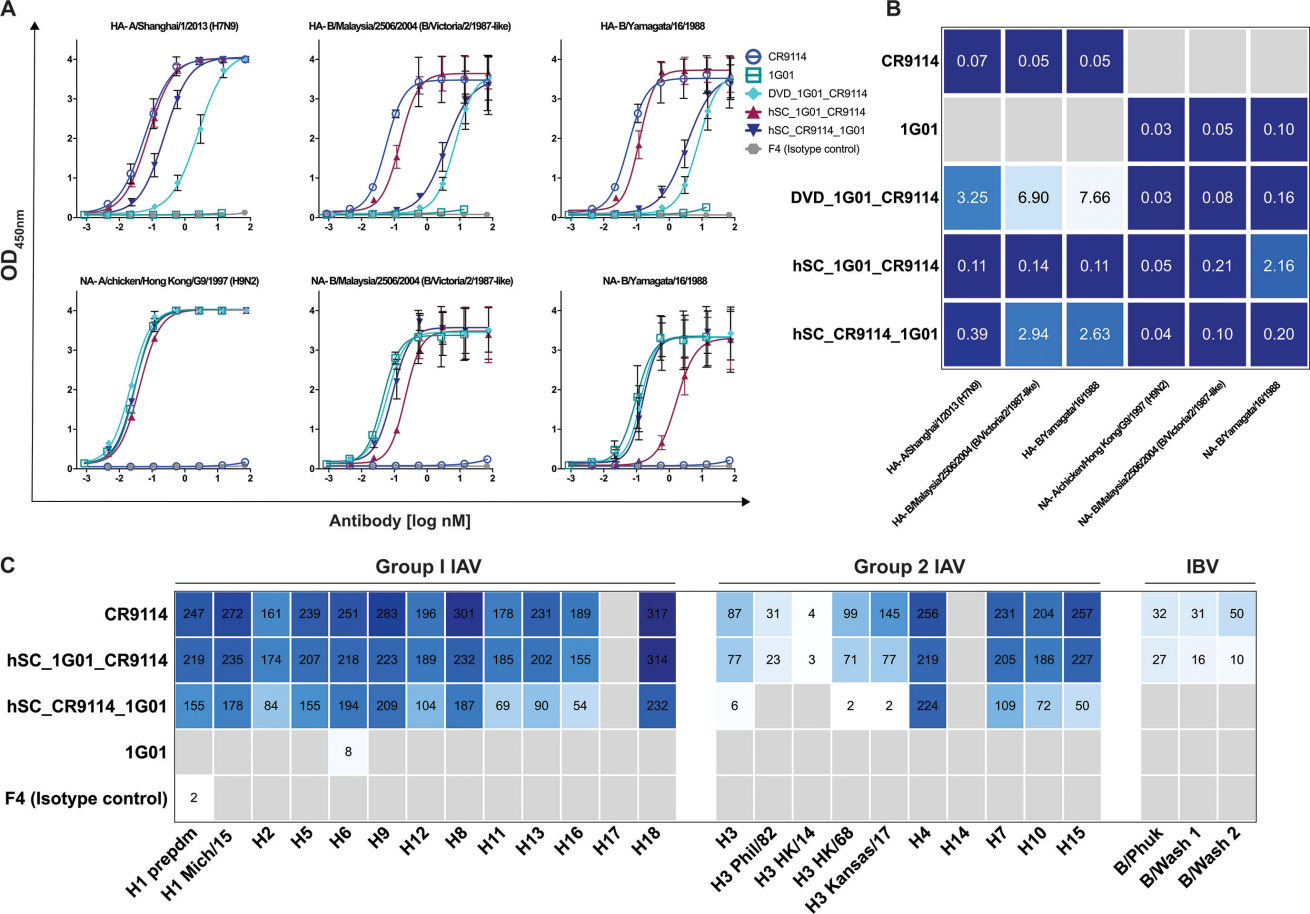
Reactivity profiles of bsAbs. (**A**) ELISA curves of mAbs and bsAbs against HA and NA antigens. (**B**) EC_50_ values (in nM) derived from curve fitting, with stronger reactivity (lower EC_50_) denoted as darker blue. (**C**) Microarray reactivities (area under the curve, AUC) of mAbs and bsAbs toward group 1 and 2 IAV, as well as IBV HAs (a full list of protein names is included in Table S3). Reactivity values are shown, with darker blue indicating stronger reactivity. Gray squares indicate no reactivity.

We examined the enzyme-linked immunosorbent assay (ELISA) binding reactivities of the three bsAbs (as well as parental mAbs) toward HA and NA from several different influenza viruses: HA (from A/Shanghai/1/2013 H7N9) and NA (from A/chicken/HongKong/G9/1997 H9N2), B/Malaysia/2506/2004 (B/Mal), and B/Yamagata/16/1988 (B/Yam) (Fig. 2A). Experiments were performed with a fixed amount of HA or NA immobilized on the wells and titrated amounts of bsAb or mAb. Parental mAbs CR9114 and 1G01 showed strong reactivity as expected (low nanomolar EC_50_ values, Fig. 2B) to their respective H7 and N2 antigens, with no cross-reactivity toward the other antigen. Additionally, CR9114 bound strongly to HA from B/Mal and B/Yam and 1G01 to NA from the same IBV strains as has been previously observed. We found that DVD_1G01_CR9114 was able to bind well to all NA types tested with low 50% effective concentration (EC_50_) values of 0.03, 0.08, and 0.16 nM to N2, B/Mal NA, and B/Yam NA, respectively, similar to 1G01. However, when compared to CR9114, DVD_CR9114_1G01 showed weaker binding to HA with significantly higher EC_50_ values against all three antigens tested. Both hSC bsAbs displayed strong cross-reactivity across HA and NA antigens from all species tested. Although EC_50_ values were higher than the parental mAbs for hSC_1G01_CR9114 toward B/Yam NA and for hSC_CR9114_1G01 toward B/Mal HA and B/Yam HA, they were nonetheless below 3 nM. Additionally, we assessed the binding reactivity of the stored samples from day 10 and 1.5 years, as well as those subjected to freeze-thaw cycles, for the parental mAbs and both hSCs against H7 and N2 antigens (Fig. S3). We found that there were little to no differences in EC_50_ values for the bsAbs between day 10 and 1.5 years and that the bsAbs retained binding activity after the freeze-thaw cycles (Table S1).

Next, we used a protein microarray coated with HA from different types of influenza viruses to further assess the binding breadth of the two hSC bsAbs (Fig. 2C). We found that both bsAbs exhibited broad reactivity toward HA from a diverse panel of group 1 and 2 IAV HA subtypes. However, in some cases, such as H2, H11, H13 (group 1), as well as H3, H10, H15 (group 2), hSC_CR9114_1G01 displayed lower reactivity than CR9114, whereas in other cases (H3 of A/Philippines/2/1982 and A/Hong Kong/4801/2014, and all IBVs), this bsAb had no activity. This lower activity may be due to the position of the CR9114 Fv in this specific format (as the scFvs in the hinge), where the CR9114 paratope may be blocked sterically by the adjacent 1G01 Fab, thereby lowering affinity against more divergent HA types.

**Fig 3:**
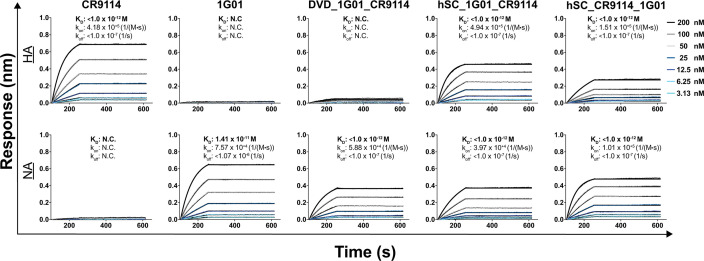
Biolayer interferometry. Binding sensorgrams against HA (A/Shanghai/1/2013 [H7N9]) and NA (A/chicken/HongKong/G9/1997 [H9N2]) at mAb/bsAb concentrations ranging from 3.13 to 200 nM. The kinetic parameters are indicated above each curve.

The binding kinetics of the bsAbs toward H7 and N2 were assessed by biolayer interferometry (BLI, Fig. 3). The bsAbs were immobilized onto the sensor and then dipped into buffer containing different dilutions of either antigen. In general, the sensorgrams could be described well with a 1:1 binding model. However, because the bsAbs are bivalent toward either antigen and HA and NA are trimeric and tetrameric, respectively, the binding stoichiometries are likely to be more complicated than a simple 1:1 model. Thus, we report “apparent” binding affinities (*K*_*D*_^app^) as we cannot rule out potential avidity effects. Nonetheless, the *K*_*D*_^app^ values are useful for comparative analysis among the bsAbs or for comparison to parental mAbs.

Consistent with the ELISA reactivity profiles, we observed that the parental mAbs bound strongly and specifically to their respective antigens with subpicomolar (*K*_*D*_^app^ < 1.0 pM CR9114 for HA) or picomolar (*K*_*D*_^app^ = 14 pM 1G01 for NA) affinities. For both mAbs, the low *K*_*D*_^app^ values resulted from slow off rates, as is common for high-affinity antibodies. These values were lower than the previously reported affinities (1.2 nM for CR9114 and 2.2 nM for 1G01), possibly due to the above-described avidity effects or the uncertainties of fitting very slow off rates. No binding was observed for 1G01 to HA or CR9114 to NA, as expected. DVD_1G01_CR9114 displayed subpicomolar binding affinity to NA (<1.0 pM), similar to 1G01; however, no binding response was observed toward HA. This lack of binding between DVD_1G01_CR9114 and HA may be due to the position of the CR9114 Fvs in the DVD-Ig format, where the “outer” 1G01 Fvs may obstruct the binding ability of the “inner” CR9114 Fvs. While some binding of DVD_1G01_CR9114 toward HAs was observed by ELISA (Fig. 2B), the EC_50_ values were ~50- to 100-fold higher than those of CR9114, indicating a lower affinity that was not detected here by BLI. We found that both hSCs have parent-like binding affinities to both HA and NA (<1.0 pM). Given these results, we focused subsequent experiments on the hSC format bsAbs.

### Neutralization, inhibition of viral entry, and inhibition of viral egress

**Fig 4:**
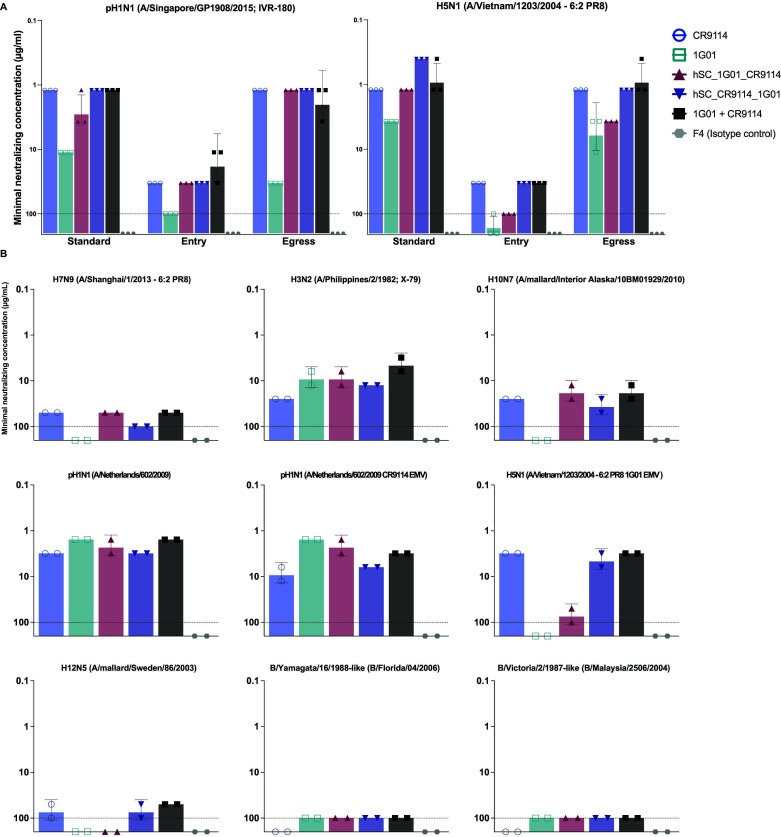
Neutralization profiles of bsAbs. (**A**) “Standard, entry, and egress” neutralizing activity against pH1N1 and H5N1 IAV. (**B**) “Standard” neutralizing activity against a panel of IAV and IBV strains, including CR9114 escape mutant virus (EMV) and 1G01 EMV, two mAb-resistant strains. The limit of detection is 100 µg/mL (indicated by the dotted line in histograms).

We next characterized the *in vitro* functionality of hSC_1G01_CR9114 and hSC_CR9114_1G01 in comparison to the parental mAbs as well as a cocktail of the mAbs in microneutralization (MN) assays (Fig. 4A). The “cocktail” concentrations reflected each of the two mAbs, and, therefore, the cocktail has twice the total mAb weight concentration of the bsAbs. Although the EC_50_ and *K*_*D*_^app^ values above are in molar antibody concentrations, the minimum neutralizing concentrations here are reported in weight concentrations to allow for comparison with previously reported assays (29). For these experiments, we added mAbs to both the inoculum and overlay (“standard”), which tests inhibitory effects on viral attachment, entry, and egress; or we added mAbs either only to the inoculum to determine effects on viral entry or only to the overlay to determine effects on viral egress. Both bsAbs had similar activity, represented by comparable minimal neutralizing concentrations (µg/mL), to the stronger of the two parental mAbs in “standard, entry, and egress” MN assays against both H1N1 and H5N1 viruses. Under all conditions, hSC_CR9114_1G01 was an equally potent neutralizer of H1N1 as CR9114, even though there was a lower molar amount of hSC_CR9114_1G01. We observed a small decrease in potency by hSC_1G01_CR9114 against H1N1 in the standard condition relative to CR9114, but this bsAb was still better than 1G01. As for H5N1, the bsAbs again neutralized similarly to the stronger parental mAb and the cocktail of the parental mAbs even though bsAbs were present in lower molar concentrations. It is noteworthy that hSC_CR9114_1G01 had a minimal neutralizing concentration three- and twofold lower than the stronger parental mAb (CR9114) and the cocktail (which had twice the amount of Ab), respectively, in the standard H5N1 MN assay. The low levels of aggregate present in antibody preparations (Fig. S2) may affect the minimal neutralizing values, but overall, our results indicate that bsAbs have similar or slightly improved activity relative to the strongest neutralizing parental mAb as well as cocktail. Precise quantification of any neutralization advantage the bsAbs may confer would require the removal of the aggregates by SEC.

We further characterized the standard neutralizing potency of the bsAbs against different influenza virus strains (Fig. 4B). We again found that the bsAbs had activity similar to the stronger parental mAb and the cocktail. Included in this panel were CR9114- and 1G01-resistant variants: CR9114 and 1G01 escape mutant viruses (EMVs). When tested against the resistant variants, hSC_1G01_CR9114 neutralized CR9114 EMV more potently than hSC_CR9114_1G01, although the latter was roughly equivalent in potency to CR9114, which retained some activity against this variant. BsAb hSC_CR9114_1G01 was able to neutralize a mutant H5N1 A/PR/8/1934-based vaccine strain that fully evaded inhibition by mAb 1G01, whereas hSC_1G01_CR9114 was not. Finally, we examined whether the bsAbs neutralized IBV strains, but they did not. This result was expected because neither CR9114 nor 1G01 can neutralize many IBVs effectively, although they are able to provide *in vivo* protection.

To further explore the potential mechanisms for inhibition and protection, we examined the NA inhibitory activity (Fig. S4) as well as antibody-dependent cellular cytotoxicity (ADCC) (Fig. S5) of the bsAbs relative to the cocktail. We found that the NA inhibitory activity of both bsAbs was as potent as 1G01 and more potent than the cocktail. ADCC of bsAbs against cells infected with three different viruses and two EMVs was reduced in comparison to the cocktail but nonetheless detectable.

### *In vivo* efficacy

**Fig 5:**
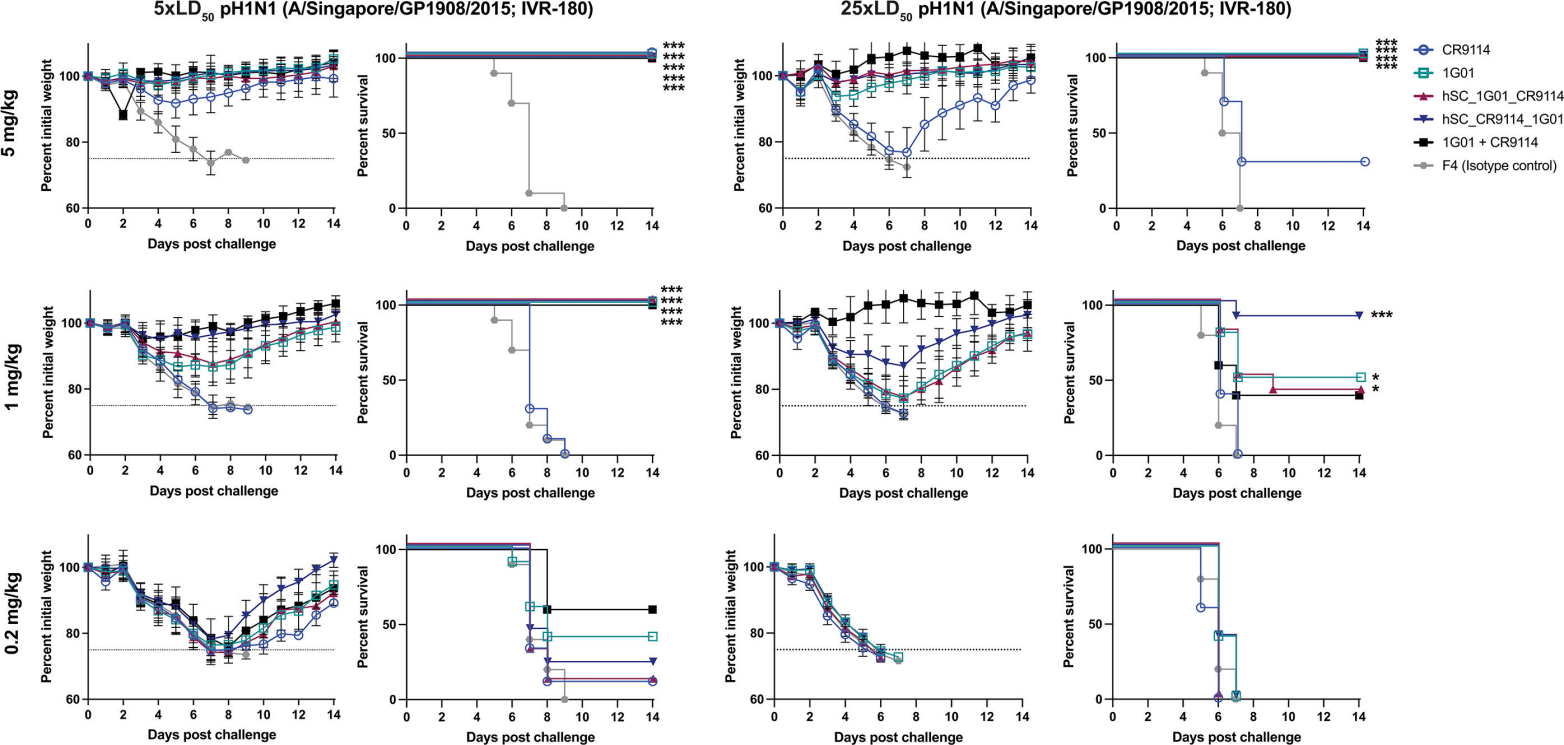
Prophylactic studies. Mice were dosed with mAb or bsAb as indicated and then challenged intranasally with 5× or 25× LD_50_ of IAV A/Singapore/GP1908/2015 (IVR-180; pH1N1). Experiments were performed twice independently with two different groups (*n* = 5) and then the data were combined, except for the cocktail (1G01 + CR9114), which consisted of a single group of *n* = 5 mice performed once. An irrelevant Sudan virus-specific mAb (F4) was used as a control (***P < 0.001 and *P < 0.05, relative to F4).

We investigated the protective capacity of the bsAbs (and parental mAbs) in both prophylactic and therapeutic settings against an H1N1 virus (A/Singapore/GP1908/2015; IVR-180). Both morbidity (weight loss) and mortality (survival) were monitored. For prophylactic studies, 6–8-week-old female BALB/c mice (*n* = 10 mice/group, except cocktail group *n* = 5) received three different doses of bsAb or mAb (0.2, 1, or 5 mg/kg) and were then challenged with either 5× or 25× the 50% lethal dose (LD_50_) of A/Singapore/GP1908/2015 (IVR-180) intranasally (Fig. 5).

In the 5× LD_50_ challenge, both the bsAbs and parental mAbs were fully protective against both weight loss and lethality at the 5 mg/kg dose; but at 1 mg/kg, only the bsAbs, 1G01, and the cocktail conferred complete survival. Furthermore, bsAb hSC_CR9114_1G01-treated mice did not experience as much weight loss as the other treatment groups at this dose. At the 0.2 mg/kg dose, both bsAbs conferred limited survival; interestingly, mice given hSC_CR9114_1G01 exhibited the least weight loss of any group. Mice provided with isotype control all succumbed to infection by day 9, with concomitant weight loss during that period.

In the 25× LD_50_ challenge, both the bsAbs, 1G01, and the cocktail conferred strong protection from morbidity and mortality, but CR9114 provided very limited protection at the highest dose (5 mg/kg) and was not protective at the lower doses (similar to isotype control mAb). When the treatment dose was reduced to 1 mg/kg, only hSC_CR9114_1G01 afforded high protection from mortality, with mice experiencing some weight loss. While hSC_1G01_CR9114 was as protective as 1G01, those groups and the cocktail group were only partially protected from mortality and suffered significant weight loss through day 7.

**Fig 6:**
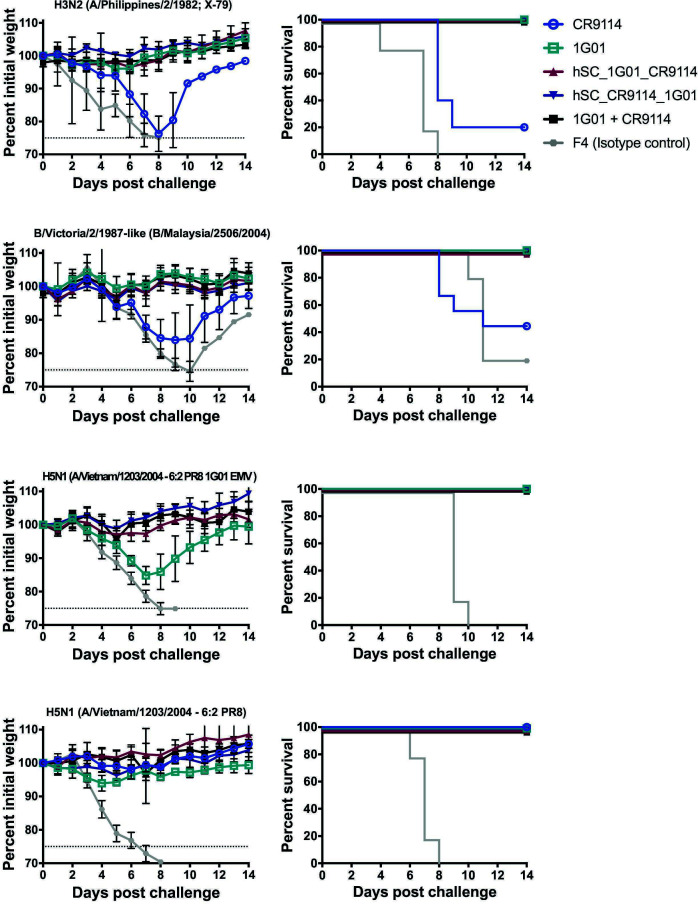
*In vivo* cross-protection studies. Mice were dosed with 5 mg/kg of mAb or bsAb as indicated and then challenged intranasally with 5× LD_50_ of diverse viruses: H3N2 (A/Philippines/2/1982; A/PR/8/1934 reassortant X-79), B/Malaysia/2506/2004 (B/Victoria/2/87-like), H5N1 1G01 EMV (A/Vietnam/1203/2004-low pathogenic 6:2 A/PR/8/1934 reassortant strain), and H5N1 (A/Vietnam/1203/2004-low pathogenic 6:2 A/PR/8/1934 reassortant strain). Experiments were performed with *n* = 5 per group. An irrelevant Sudan virus-specific mAb (F4) was used as a control.

To explore the breadth of protection, we performed smaller cross-protection studies (*n* = 5) where we dosed the mice with 5 mg/kg of each antibody and then challenged the mice intranasally with 5× LD_50_ of diverse viruses (Fig. 6). When the mice were challenged with H3N2 (A/Philippines/2/1982; X-79), we observed that the CR9114 group experienced high morbidity, and only 20% of the mice survived. However, mice given either of the bsAbs showed little to no morbidity and 100% survival. We observed a similar trend of high morbidity and low survival rates for mice given CR9114 and challenged with B/Malaysia/2506/2004, whereas the two bsAbs again showed little to no sickness and provided 100% survival as did the cocktail group. Then, we sought to test the level of broad protection the bsAbs have by challenging mice with EMVs, H5N1 1G01 EMV (based on the A/Vietnam/1203/2004-low pathogenic 6:2 A/PR/8/1934 vaccine strain), and the respective non-mutated version of this virus. Interestingly, though all mice infected with H5N1 1G01 EMV and given 1G01 survive, these mice do experience high levels of weight loss, thus high morbidity. More importantly, mice infected with H5N1 1G01 EMV and given either of our bsAbs show minimum weight loss, indicating that bsAbs could better protect against morbidity than 1G01. These mice also had a 100% survival rate. Even though none of the bsAbs outperformed the cocktail group (with 2× the amount of mAb), our data show that the bsAbs were better at preventing the progression of the disease, compared to CR9114 and 1G01, and they provided high levels of protection, unlike CR9114, against diverse influenza viruses.

**Fig 7:**
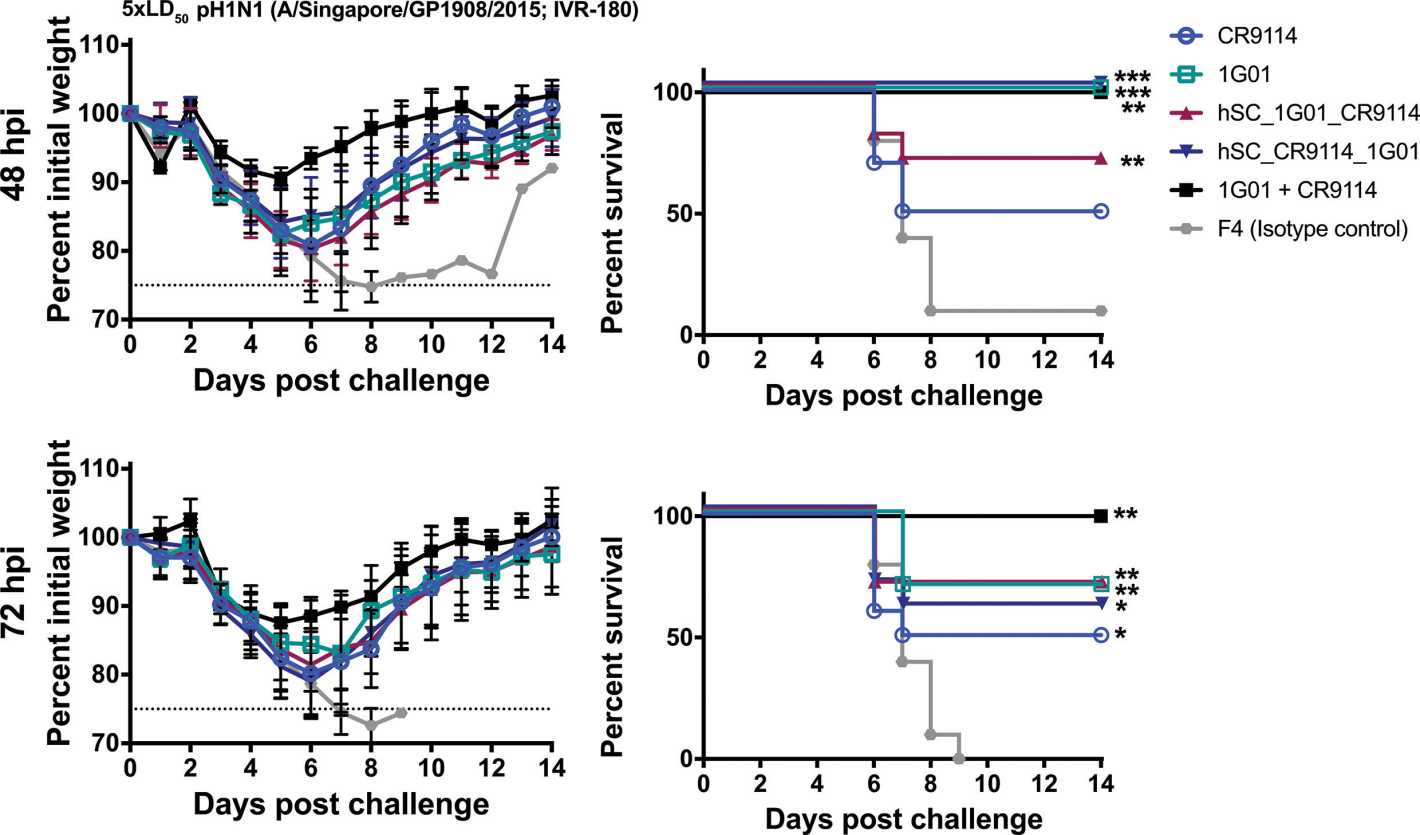
Therapeutic studies. Mice were challenged with 5× LD_50_ A/Singapore/GP1908/2015 (IVR-180; pH1N1) and then dosed 48 or 72 hpi at 5 mg/kg bsAb/mAb or 10 mg/kg cocktail. Experiments were performed twice independently with two different groups (*n* = 5) and then the data were combined, except for the cocktail (1G01 + CR9114), which consisted of a single group of *n* = 5 mice performed once. An irrelevant Sudan virus-specific mAb (**F4**) was used as a control (***P < 0.001, **P < 0.01, and *P < 0.05, relative to F4).

Mice were similarly challenged with 5× LD_50_ of virus and then treated with 5 mg/kg of bsAbs or mAbs 48- or 72 hours post-infection (hpi) to examine the therapeutic potential of the Abs (Fig. 7). For the cocktail, the 5 mg/kg represents each component, and therefore the total mAb dose was 10 mg/kg, twice that of the bsAb. With the 48 hpi treatment, only hSC_CR9114_1G01, 1G01, and the cocktail were completely protective against lethality; hSC_1G01_CR9114 was partially protective, more so than CR9114. All groups experienced weight loss. When treatment was delayed to 72 hours, both bsAbs, CR9114, and 1G01 were partially protective against mortality but not morbidity, while the cocktail group had complete survival. Together, these results indicate that bsAb hSC_CR9114_1G01 has advantages over either monotherapy under some dosing/challenge settings, which could be beneficial, particularly with stringent prophylactic dosing.

Finally, we monitored the pharmacokinetics of the antibodies *in vivo* for 35 days and found that the half-life of hSC_CR9114_1G01 (*t*_1/2_: 9.6 days) was comparable to mAbs CR9114 and 1G01(*t*_1/2_: 6.1 and 12.2 days, respectively). The half-life of hSC_1G01_CR9114 was lower (*t*_1/2_: 4.0 days) but still within the range of most mAbs (Fig. S6).

## DISCUSSION

Here, we describe the design and evaluation of novel HA/NA dual-targeting influenza virus bsAbs. The initial panel included DVD-Ig, hSC, and scFv-Ig formats, with different Fv domain organizations. However, we found that both scFv-Igs, as well as DVD_CR9114_1G01, were not amenable to large-scale expression. Furthermore, DVD_1G01_CR9114 retained parent-like NA activity, but HA activity was significantly lower than CR9114. This binding profile may have resulted from steric occlusion of the CR9114 Fvs by the outer set of 1G01 Fvs. The basis for poor expression/stability profiles of the other bsAbs is unknown, but we have previously found that certain Fvs are less amenable to integration into some bsAb formats (34). Nonetheless, the hSC formats were expressible, retained binding activity to both antigens, and were protective *in vivo*. A potential future direction is to examine other bsAb formats, including “asymmetric” designs, where each Fab arm has a different specificity (e.g., Duobody) (37). Although such molecules would be monovalent toward each antigen (as opposed to bivalent, as is the case with each of the formats tested here), the integration of each set of Fvs into a Fab may improve the stability and accessibility of the paratope.

The hSC bsAbs were roughly equivalent to the cocktail, albeit at lower molar concentrations, as well as CR9114, the more potent neutralizer of the two parental mAbs, in neutralization potency and breadth. These results imply that the synergistic neutralization advantage to the direct physical linkage of 1G01 and CR9114 is modest as the bsAbs were equally neutralizing at half the molar concentration as the cocktail. In the case of CCHFV bsAbs, we found that synergistic neutralization was enhanced with DVD-Igs relative to cocktails, although this outcome was largely empirical because the bsAbs were not designed with any specific structural hypothesis (e.g., distance between the two paratopes) in mind (26). bsAb hSC_CR9114_1G01 maintained its ability to neutralize a 1G01-resistant strain, and thus one advantage conferred is a lower susceptibility to viral escape in a single molecule. Similar activities have been noted in human immunodeficiency virus 1 (HIV-1) multispecific mAbs (38–40).

*In vivo* studies revealed that hSC bsAbs are highly protective and may even confer an advantage relative to a parental cocktail under some prophylactic dosing conditions. We note that the ADCC activity observed for both CR9114 and bsAbs was lower than expected *in vitro*, relative to previous reports, and thus the protection here likely relies heavily on the neutralizing activity. The discrepancy in ADCC activity may be due to two mutations (R214K and G237R) present in the CH1 domain of the pMAZ-IgH plasmid, relative to the most common IgG1 allotype (IGHG1*03) (Fig. S7). Although we have not tested the specific binding of bsAbs to Fcγ receptors, the G237R mutation in particular is proximal to L234/L235, which are key mediators of ADCC-specific FcγR interactions (41). Alternatively, ADCC assay conditions or bsAb Fc glycosylation patterns may also contribute. Again, any synergistic advantages of the bsAbs relative to the cocktail are subtle (bsAbs were dosed at lower molar concentrations than the cocktail), but it is nonetheless noteworthy that bsAbs have strong prophylactic and therapeutic activity and therefore have strong immunotherapeutic potential. Another possible advantage of bsAbs would be an increased resistance to the development of viral escape *in vivo*, but this was not tested here. We describe new dual-targeting HA/NA bsAbs with prophylactic and therapeutic protective properties. This work provides insight into the design of influenza bsAb immunotherapies.

## MATERIALS AND METHODS

### Cells, viruses, and proteins

Madin Darby canine kidney (MDCK) (CCL-34, ATCC) cells were grown and maintained in Dulbecco’s modified Eagle’s medium supplemented with 10% fetal bovine serum (FBS), 1% 4-(2-hydroxyethyl)-1-piperazineethanesulfonic acid (HEPES), and 100 U/mL penicillin and 100 µg/mL streptomycin (pen-strep). Sf9 (*Spodoptera frugiperda*) cells were maintained in *Trichoplusia ni* medium formulation Hinkel medium (Gemini Bio-Products) supplemented with 10% FBS, 0.1% pluronic F68, and pen-strep. ADCC bioeffector FcγRIIIa cells (Promega) were purchased as single-use aliquots and were thawed before use. Influenza A and B viruses were grown in 8–10-day-old embryonated chicken eggs (Charles River Laboratories) at 37°C for 2 days or 33°C for 3 days, respectively. Virus reassortants were rescued by plasmid-based reverse genetic techniques as previously described (42). A full list of viruses used in this study can be found in Table S2. Recombinant influenza virus HA and NA proteins were expressed in the baculovirus expression system as previously described (43). A full list of recombinant proteins used in this study can be found in Table S3. The CR9114 EMV is described in reference 44. The 1G01 escape mutant was generated in a similar manner.

### Antibody expression and purification

Gene fragments with heavy and light chains of 1G01 and CR9114 in different arrangements designed to make the DVDs, SCs, and hSCs were sub-cloned into the respective pMAZ- IgH and pMAZ-IgL vectors developed by Mazor et al. (45). Subsequently, the respective pMAZ-IgH and pMAZ-IgL vectors encoding each antibody were transiently co-transfected into ExpiCHO cells using the ExpiFectamine CHO Transfection Kit. Transfected ExpiCHO cell cultures were incubated at 37°C and 8% CO_2_ for 8 days post-transfection. To isolate antibodies, cell-free supernatants were then applied to a protein A affinity column (0.5 mL of packed beads per 25 mL of culture) (Thermo Scientific). Antibodies were purified using the Gentle Antibody Elution System (Thermo Scientific) according to the manufacturer’s instructions and then exchanged into 150 mM 4-(2-hydroxyethyl)-1-piperazineethanesulfonic acid (HEPES) and 200 mM NaCl at pH 7.4. The expression, purity, and stability of the antibodies were determined by reducing and non-reducing SDS-PAGE analysis.

### Enzyme-linked immunosorbent assay

HA and NA proteins were expressed in the baculovirus expression system as previously described (43). The HA and NA target proteins at 0.5 µg/mL in phosphate-buffered saline (PBS; pH 8.0) were directly immobilized onto 96-well EIA/RIA high binding plates (Costar) by incubating overnight for 14–16 hours at 4°C. Wells were then incubated with blocking solution (PBS [pH 7.4], 1% bovine serum albumin, BSA) for 1.5 hours at room temperature while shaking. Negative control wells were coated with 1% BSA. The antibodies were then serially diluted with PBST (PBS [pH 7.4], 1% BSA, and 0.05% Tween-20), added to the wells, and incubated for 1 hour at room temperature while shaking. Afterward, the plates were washed four to six times with PBST (PBS [pH 7.4] and 0.5% Tween-20) and incubated for 1 hour at room temperature while shaking with anti-human IgG-horseradish peroxidase (HRP) antibody conjugate (1:5,000 dilution in PBT). The wells were washed four to six times with PBST, developed using 3, 3′, 5, 5′-tetramethylbenzidine (Sigma-Aldrich, St. Louis, MO, USA) and quenched with 0.5 M sulfuric acid. The absorbance at 450 nm was determined. The data were fit to standard four-parameter logistic equations using GraphPad Prism software. The half-maximal binding titers (EC_50_) were obtained from inflection points in the curves.

### Influenza virus protein microarray

Recombinant HA proteins were spotted in arrays on Nexterion E epoxysilane-coated glass slides (Schott, Mainz, Germany). HAs were spotted in triplicate, and 24 arrays were spotted on each slide. Each HA droplet had a volume of 34 nL and was spotted onto the slides at a concentration of 100 µg/mL in 0.1% milk PBS. Slides were stored at −80°C in vacuum-sealed slide boxes until use. On the day of the assay, slides were allowed to warm to room temperature before being removed from their sealed boxes, then incubated for 2 hours at >95% relative humidity at room temperature. Slides were allowed to dry and then inserted into 96-well microarray gaskets (Arrayit, Sunnyvale, CA, USA) and blocked with 3% milk in PBST for 2 hours. After the blocking solution was removed, Abs were added at a starting concentration of 30 µg/mL in 1% milk PBST at a volume of 100  µL/array, and two 10-fold dilutions were performed across each slide. After a 1-hour incubation, slides were washed three times with 220 µL/array PBST before the addition of 100 µL of secondary antibody solution, comprising Cy5-labeled anti-human IgG secondary antibody diluted 1:1,000 in 1% milk PBST. After 1 hour, the secondary antibody solution was removed, and the arrays were washed three times with 220 µL/array PBST, removed from the 96-well microarray gaskets, rinsed with deionized water, and dried with an air compressor. Microarray slides were analyzed with a Vidia microarray scanner (Indevr, Boulder, CO, USA) at an exposure time of 1,000 ms, and AUC was calculated from median fluorescence as a total peak area above a fluorescence of 0.04.

### Biolayer interferometry

The OctetRed system (ForteBio, Pall LLC) was used to determine the binding interactions of the mAbs, DVDs, SCs, and hSCs to both HA (A/Shanghai/1/2013 [H7N9]) and NA (A/chicken/HongKong/G9/1997 [H9N2]). Anti-human IgG Fc sensors were used for initial antibody loading and then incubated with several different concentrations of HA or NA to determine association rates, dissociation rates, and apparent affinities (*K*_*D*_^app^). Global data fitting to a 1:1 binding model was used to assess the association rate constant, dissociation rate constant, and equilibrium dissociation constant. Data were analyzed with the Octet Data Analysis v.11 software, and at least four curves were globally fitted.

### Enzyme-linked lectin assay

To determine the neuraminidase activity of the viruses, 96-well microtiter plates (Thermo Fisher) were coated with 100 µL fetuin (Sigma) at a concentration of 25 µg/mL in PBS at 4°C overnight. The plates were blocked with Blotto blocking buffer (Thermo Scientific) for 1 hour at 37°C. Twofold serial dilutions of viruses were added, and the plates were incubated overnight at 37°C. The plates were washed and 100 µL of HRP-conjugated peanut agglutinin (5 µg/mL) was added, and the plates were further incubated for 1 hour. Following incubation, the plates were washed and developed with 100 µL of SigmaFast OPD. The reaction was stopped after 10 minutes with 100 µL of 3 M hydrochloric acid (Thermo Fisher), and the absorbance was measured at a wavelength of 490 nm using a microtiter plate reader (Bio-Tek). The 50% effective concentration (EC_50_) was calculated using GraphPad Prism (version 9).

For the neuraminidase inhibition assays, microtiter plates were coated with fetuin and blocked as previously described. Fifty microliters of threefold serially diluted Abs at a starting concentration of 30 µg/mL were mixed with an equal volume of diluted viruses (2× EC_50_) for 1 hour. The fetuin-coated plates were washed, and the virus/Ab mixture was added to the plates and incubated overnight at 37°C. The assay was further carried out as described above. The data were analyzed using GraphPad Prism (version 9).

### ADCC reporter bioassay

MDCK cells were infected with 100 µL of virus at a multiplicity of infection of 5 in UltraMDCK medium (Lonza) and incubated for 24 hours at 37°C. Abs were threefold diluted to a starting concentration of 30 µg/mL in RPMI 1640 medium (Life Technologies). A volume of 25 μL of serially diluted Abs, 25 µL human ADCC bioeffector FcγRIIIa cells (Promega) (6 × 10^6^ cells/mL), and 25 µL of RPMI 1640 medium were added to each well. Following a 6-hour incubation at 37°C, 75 µL of Bio-Glo luciferase (Promega) was added to each well. The plates were incubated for 10 minutes, and luminescence was measured with a microplate reader (BioTek). The results were analyzed using GraphPad Prism (version 9).

### Microneutralization assay

Abs were twofold serially diluted in infection medium (UltraMDCK medium [Lonza] supplemented with 1 µg/mL tosyl phenylalanyl chloromethyl ketone-treated trypsin [infection media; Sigma]) starting at 100  µg/mL, and 50 µL were mixed with 50 µL (100 TCID_50_) of diluted virus in the same medium. Following a 1-hour incubation, the virus/Ab mixture (or virus only for egress conditions) was added to a monolayer of MDCK cells in 96-well plates and further incubated for 1 hour. The mixture was then removed, and the cells were further incubated in infection medium (entry conditions) or Ab (standard and egress conditions) at 37°C for 48 hours (for IAV) or at 33°C for 72 hours (for IBV), and virus growth was detected using a classical hemagglutination assay. The minimal inhibitory concentration was determined as the highest antibody dilution capable of neutralizing the virus.

### Antibody protection experiments in mice

Protection experiments were carried out to assess the prophylactic and therapeutic efficacy of the Abs against lethal influenza virus challenges. Female 6–8-week-old BALB/c mice (*n* = 10 mice/group, except cocktail group *n* = 5/group) were used for those experiments. For the dose escalation studies, mice were injected intraperitoneally (ip) with 100 µL of Ab at a concentration of 5, 1, and 0.2 mg/kg (cocktail groups were injected with 2× concentration) 2 hours prior to virus challenges with either 5× LD_50_ or 25× LD_50_ of pH1N1 (A/Singapore/GP1908/2015; IVR-180). For the therapeutic setting, mice were infected with 5× LD_50_ of pH1N1 (A/Singapore/GP1908/2015; IVR-180). The Abs were administered (100 µL ip) 48 or 72 hpi at a concentration of 5 mg/kg (cocktail groups were injected with 2× concentration). For the prophylactic setting, Abs (5 mg/kg) were administered (100 µL ip) 2 hours prior to virus challenges, mice (*n* = 5/group) were then infected with 5× LD_50_ of H3N2 (A/Philippines/2/1982; 6:2 A/PR/8/1934 reassortant X-79), B/Malaysia/2506/2004 (B/Victoria/2/1987-like), H5N1 1G01 EMV (A/Vietnam/1203/2004-low pathogenic 6:2 A/PR/8/1934 reassortant vaccine strain), and H5N1 (A/Vietnam/1203/2004-low pathogenic 6:2 A/PR/8/1934 reassortant vaccine strain). The percentage of survival and weight change following the challenge were monitored for 14 days. Mice that experienced a body weight reduction of greater than 25% were humanely euthanized and scored dead. Fisher’s Exact test was utilized to compare statistical differences between treatment groups.

### *In vivo* half-life studies

The *in vivo* half-life of the bsAbs and parental mAbs were assessed in mice. Female 6–8-week-old BALB/c mice (*n* = 5 mice/group) were used for those experiments. Antibodies were administered ip at a 5 mg/kg dose (in the case of the mAb cocktail, 5 mg/kg of each mAb), and serum samples were collected on days 0, 1, and every week following until 5 weeks. The amount of mAb present in the serum was measured using a Human IgG ELISA Kit (Millipore Sigma) following the manufacturer’s instructions. The results were analyzed using GraphPad Prism (version 9).

## SUPPLEMENTAL MATERIAL

Supplemental material mbio.01085-24-s0001.docx Supplemental figures and tables.

ASM does not own the copyrights to Supplemental Material that may be linked to, or accessed through, an article. The authors have granted ASM a non-exclusive, world-wide license to publish the Supplemental Material files. Please contact the corresponding author directly for reuse.

## References

[1] Gamblin SJ, Skehel JJ. 2010. Influenza hemagglutinin and neuraminidase membrane glycoproteins. J Biol Chem 285:28403–28409. doi:10.1074/jbc.R110.129809 20538598 PMC2937864

[2] Du R, Cui Q, Rong L. 2019. Competitive cooperation of hemagglutinin and neuraminidase during influenza A virus entry. Viruses 11:458. doi:10.3390/v11050458 31137516 PMC6563287

[3] Krammer F, García-Sastre A, Palese P. 2018. Is it possible to develop a “universal” influenza virus vaccine? potential target antigens and critical aspects for a universal influenza vaccine Cold Spring Harb Perspect Biol 10:a028845. doi:10.1101/cshperspect.a028845 28663209 PMC6028071

[4] Krammer F, Smith GJD, Fouchier RAM, Peiris M, Kedzierska K, Doherty PC, Palese P, Shaw ML, Treanor J, Webster RG, García-Sastre A. 2018. Influenza. Nat Rev Dis Primers 4:3. doi:10.1038/s41572-018-0002-y 29955068 PMC7097467

[5] Prachanronarong KL, Canale AS, Liu P, Somasundaran M, Hou S, Poh Y-P, Han T, Zhu Q, Renzette N, Zeldovich KB, Kowalik TF, Kurt-Yilmaz N, Jensen JD, Bolon DNA, Marasco WA, Finberg RW, Schiffer CA, Wang JP. 2019. Mutations in influenza A virus neuraminidase and hemagglutinin confer resistance against a broadly neutralizing hemagglutinin stem antibody. J Virol 93:e01639-18. doi:10.1128/JVI.01639-18 30381484 PMC6321927

[6] Tharakaraman K, Subramanian V, Cain D, Sasisekharan V, Sasisekharan R. 2014. Broadly neutralizing influenza hemagglutinin stem-specific antibody CR8020 targets residues that are prone to escape due to host selection pressure. Cell Host Microbe 15:644–651. doi:10.1016/j.chom.2014.04.009 24832457 PMC4258880

[7] Powell H, Pekosz A. 2020. Neuraminidase antigenic drift of H3N2 clade 3c.2a viruses alters virus replication, enzymatic activity and inhibitory antibody binding. PLoS Pathog 16:e1008411. doi:10.1371/journal.ppat.1008411 32598381 PMC7351227

[8] Schmidt AG, Therkelsen MD, Stewart S, Kepler TB, Liao H-X, Moody MA, Haynes BF, Harrison SC. 2015. Viral receptor-binding site antibodies with diverse germline origins. Cell 161:1026–1034. doi:10.1016/j.cell.2015.04.028 25959776 PMC4441819

[9] Sakai T, Nishimura SI, Naito T, Saito M. 2017. Influenza A virus hemagglutinin and neuraminidase act as novel Motile machinery. Sci Rep 7:45043. doi:10.1038/srep45043 28344335 PMC5366856

[10] Guo H, Rabouw H, Slomp A, Dai M, van der Vegt F, van Lent JWM, McBride R, Paulson JC, de Groot RJ, van Kuppeveld FJM, de Vries E, de Haan CAM. 2018. Kinetic analysis of the influenza A virus HA/NA balance reveals contribution of NA to virus-receptor binding and NA-dependent rolling on receptor-containing surfaces. PLoS Pathog 14:e1007233. doi:10.1371/journal.ppat.1007233 30102740 PMC6107293

[11] Vahey MD, Fletcher DA. 2019. Influenza A virus surface proteins are organized to help penetrate host mucus. Elife 8:e43764. doi:10.7554/eLife.43764 31084711 PMC6516830

[12] Tharmalingam T, Han X, Wozniak A, Saward L. 2022. Polyclonal hyper immunoglobulin: a proven treatment and prophylaxis platform for passive immunization to address existing and emerging diseases. Hum Vaccin Immunother 18:1886560. doi:10.1080/21645515.2021.1886560 34010089 PMC9090292

[13] Burton DR, Poignard P, Stanfield RL, Wilson IA. 2012. Broadly neutralizing antibodies present new prospects to counter highly antigenically diverse viruses. Science 337:183–186. doi:10.1126/science.1225416 22798606 PMC3600854

[14] Julien JP, Lee PS, Wilson IA. 2012. Structural insights into key sites of vulnerability on HIV-1 Env and influenza HA. Immunol Rev 250:180–198. doi:10.1111/imr.12005 23046130 PMC3479221

[15] Kwong PD, Wilson IA. 2009. HIV-1 and influenza antibodies: seeing antigens in new ways. Nat Immunol 10:573–578. doi:10.1038/ni.1746 19448659 PMC2796958

[16] Wec AZ, Bornholdt ZA, He S, Herbert AS, Goodwin E, Wirchnianski AS, Gunn BM, Zhang Z, Zhu W, Liu G, et al.. 2019. Development of a human antibody cocktail that deploys multiple functions to confer pan-ebolavirus protection. Cell Host Microbe 25:39–48. doi:10.1016/j.chom.2018.12.004 30629917 PMC6396299

[17] Wec AZ, Herbert AS, Murin CD, Nyakatura EK, Abelson DM, Fels JM, He S, James RM, de La Vega M-A, Zhu W, Bakken RR, Goodwin E, Turner HL, Jangra RK, Zeitlin L, Qiu X, Lai JR, Walker LM, Ward AB, Dye JM, Chandran K, Bornholdt ZA. 2017. Antibodies from a human survivor define sites of vulnerability for broad protection against Ebolaviruses. Cell 169:878–890. doi:10.1016/j.cell.2017.04.037 28525755 PMC5808922

[18] Barba-Spaeth G, Dejnirattisai W, Rouvinski A, Vaney M-C, Medits I, Sharma A, Simon-Lorière E, Sakuntabhai A, Cao-Lormeau V-M, Haouz A, England P, Stiasny K, Mongkolsapaya J, Heinz FX, Screaton GR, Rey FA. 2016. Structural basis of potent Zika-dengue virus antibody cross-neutralization. Nature 536:48–53. doi:10.1038/nature18938 27338953

[19] Dejnirattisai W, Wongwiwat W, Supasa S, Zhang X, Dai X, Rouvinski A, Jumnainsong A, Edwards C, Quyen NTH, Duangchinda T, Grimes JM, Tsai W-Y, Lai C-Y, Wang W-K, Malasit P, Farrar J, Simmons CP, Zhou ZH, Rey FA, Mongkolsapaya J, Screaton GR. 2015. A new class of highly potent, broadly neutralizing antibodies isolated from viremic patients infected with dengue virus. Nat Immunol 16:170–177. doi:10.1038/ni.3058 25501631 PMC4445969

[20] Kim AS, Kafai NM, Winkler ES, Gilliland TC, Cottle EL, Earnest JT, Jethva PN, Kaplonek P, Shah AP, Fong RH, Davidson E, Malonis RJ, Quiroz JA, Williamson LE, Vang L, Mack M, Crowe JE, Doranz BJ, Lai JR, Alter G, Gross ML, Klimstra WB, Fremont DH, Diamond MS. 2021. Pan-protective anti-alphavirus human antibodies target a conserved E1 protein epitope. Cell 184:4414–4429. doi:10.1016/j.cell.2021.07.006 34416146 PMC8382027

[21] Malonis RJ, Earnest JT, Kim AS, Angeliadis M, Holtsberg FW, Aman MJ, Jangra RK, Chandran K, Daily JP, Diamond MS, Kielian M, Lai JR. 2021. Near-germline human monoclonal antibodies neutralize and protect against multiple arthritogenic alphaviruses. Proc Natl Acad Sci USA 118:37. doi:10.1073/pnas.2100104118 PMC8449321 34507983

[22] Gunn BM, Yu W-H, Karim MM, Brannan JM, Herbert AS, Wec AZ, Halfmann PJ, Fusco ML, Schendel SL, Gangavarapu K, et al.. 2018. A role for FC function in therapeutic monoclonal antibody-mediated protection against Ebola virus. Cell Host Microbe 24:221–233. doi:10.1016/j.chom.2018.07.009 30092199 PMC6298217

[23] Frei JC, Nyakatura EK, Zak SE, Bakken RR, Chandran K, Dye JM, Lai JR. 2016. Bispecific antibody affords complete post-exposure protection of mice from both Ebola (Zaire) and Sudan viruses. Sci Rep 6:19193. doi:10.1038/srep19193 26758505 PMC4725817

[24] Wec AZ, Nyakatura EK, Herbert AS, Howell KA, Holtsberg FW, Bakken RR, Mittler E, Christin JR, Shulenin S, Jangra RK, Bharrhan S, Kuehne AI, Bornholdt ZA, Flyak AI, Saphire EO, Crowe JE, Aman MJ, Dye JM, Lai JR, Chandran K. 2016. “A "Trojan horse" bispecific-antibody strategy for broad protection against Ebolaviruses”. Science 354:350–354. doi:10.1126/science.aag3267 27608667 PMC5647781

[25] Wirchnianski AS, Wec AZ, Nyakatura EK, Herbert AS, Slough MM, Kuehne AI, Mittler E, Jangra RK, Teruya J, Dye JM, Lai JR, Chandran K. 2021. Two distinct lysosomal targeting strategies afford Trojan horse antibodies with Pan-Filovirus activity. Front Immunol 12:729851. doi:10.3389/fimmu.2021.729851 34721393 PMC8551868

[26] Fels JM, Maurer DP, Herbert AS, Wirchnianski AS, Vergnolle O, Cross RW, Abelson DM, Moyer CL, Mishra AK, Aguilan JT, et al.. 2021. Protective neutralizing antibodies from human survivors of Crimean-Congo hemorrhagic fever. Cell 184:3486–3501. doi:10.1016/j.cell.2021.05.001 34077751 PMC8559771

[27] Nyakatura EK, Soare AY, Lai JR. 2017. Bispecific antibodies for viral immunotherapy. Hum Vaccin Immunother 13:836–842. doi:10.1080/21645515.2016.1251536 27786606 PMC5425146

[28] Dreyfus C, Laursen NS, Kwaks T, Zuijdgeest D, Khayat R, Ekiert DC, Lee JH, Metlagel Z, Bujny MV, Jongeneelen M, et al.. 2012. Highly conserved protective epitopes on influenza B viruses. Science 337:1343–1348. doi:10.1126/science.1222908 22878502 PMC3538841

[29] Stadlbauer D, Zhu X, McMahon M, Turner JS, Wohlbold TJ, Schmitz AJ, Strohmeier S, Yu W, Nachbagauer R, Mudd PA, Wilson IA, Ellebedy AH, Krammer F. 2019. Broadly protective human antibodies that target the active site of influenza virus neuraminidase. Science 366:499–504. doi:10.1126/science.aay0678 31649200 PMC7105897

[30] Rajendran M, Nachbagauer R, Ermler ME, Bunduc P, Amanat F, Izikson R, Cox M, Palese P, Eichelberger M, Krammer F. 2017. Analysis of anti-influenza virus neuraminidase antibodies in children, adults, and the elderly by ELISA and enzyme inhibition: evidence for original antigenic sin. mBio 8:e02281-16. doi:10.1128/mBio.02281-16 28325769 PMC5362038

[31] Pace CS, Song R, Ochsenbauer C, Andrews CD, Franco D, Yu J, Oren DA, Seaman MS, Ho DD. 2013. Bispecific antibodies directed to CD4 domain 2 And HIV envelope exhibit exceptional breadth and picomolar potency against HIV-1. Proc Natl Acad Sci USA 110:13540–13545. doi:10.1073/pnas.1304985110 23878231 PMC3746901

[32] Shi X, Deng Y, Wang H, Ji G, Tan W, Jiang T, Li X, Zhao H, Xia T, Meng Y, Wang C, Yu X, Yang Y, Li B, Qin E-D, Dai J, Qin C-F, Guo Y. 2016. A bispecific antibody effectively neutralizes all four serotypes of dengue virus by simultaneous blocking virus attachment and fusion. MAbs 8:574–584. doi:10.1080/19420862.2016.1148850 26905804 PMC4966856

[33] Schanzer J, Jekle A, Nezu J, Lochner A, Croasdale R, Dioszegi M, Zhang J, Hoffmann E, Dormeyer W, Stracke J, Schäfer W, Ji C, Heilek G, Cammack N, Brandt M, Umana P, Brinkmann U. 2011. Development of tetravalent, bispecific CCR5 antibodies with antiviral activity against CCR5 monoclonal antibody-resistant HIV-1 strains. Antimicrob Agents Chemother 55:2369–2378. doi:10.1128/AAC.00215-10 21300827 PMC3088204

[34] Nyakatura EK, Zak SE, Wec AZ, Hofmann D, Shulenin S, Bakken RR, Aman MJ, Chandran K, Dye JM, Lai JR. 2018. Design and evaluation of bi- and trispecific antibodies targeting multiple filovirus glycoproteins. J Biol Chem 293:6201–6211. doi:10.1074/jbc.RA117.001627 29500195 PMC5912469

[35] Bezabeh B, Fleming R, Fazenbaker C, Zhong H, Coffman K, Yu X-Q, Leow CC, Gibson N, Wilson S, Stover CK, Wu H, Gao C, Dimasi N. 2017. Insertion of scFv into the hinge domain of full-length IgG1 monoclonal antibody results in tetravalent bispecific molecule with robust properties. MAbs 9:240–256. doi:10.1080/19420862.2016.1270492 27981887 PMC5297521

[36] DiGiandomenico A, Keller AE, Gao C, Rainey GJ, Warrener P, Camara MM, Bonnell J, Fleming R, Bezabeh B, Dimasi N, Sellman BR, Hilliard J, Guenther CM, Datta V, Zhao W, Gao C, Yu X-Q, Suzich JA, Stover CK. 2014. A multifunctional bispecific antibody protects against Pseudomonas aeruginosa. Sci Transl Med 6:262ra155. doi:10.1126/scitranslmed.3009655 25391481

[37] Labrijn AF, Meesters JI, de Goeij B, van den Bremer ETJ, Neijssen J, van Kampen MD, Strumane K, Verploegen S, Kundu A, Gramer MJ, van Berkel PHC, van de Winkel JGJ, Schuurman J, Parren P. 2013. Efficient generation of stable bispecific IgG1 by controlled Fab-arm exchange. Proc Natl Acad Sci USA 110:5145–5150. doi:10.1073/pnas.1220145110 23479652 PMC3612680

[38] De Gasparo R, Pedotti M, Simonelli L, Nickl P, Muecksch F, Cassaniti I, Percivalle E, Lorenzi JCC, Mazzola F, Magrì D, et al.. 2021. Bispecific IgG neutralizes SARS-Cov-2 variants and prevents escape in mice. Nature 593:424–428. doi:10.1038/s41586-021-03461-y 33767445 PMC12910678

[39] Huang Y, Yu J, Lanzi A, Yao X, Andrews CD, Tsai L, Gajjar MR, Sun M, Seaman MS, Padte NN, Ho DD. 2016. Engineered bispecific antibodies with exquisite HIV-1-neutralizing activity. Cell 165:1621–1631. doi:10.1016/j.cell.2016.05.024 27315479 PMC4972332

[40] Xu L, Pegu A, Rao E, Doria-Rose N, Beninga J, McKee K, Lord DM, Wei RR, Deng G, Louder M, et al.. 2017. Trispecific broadly neutralizing HIV antibodies mediate potent SHIV protection in macaques. Science 358:85–90. doi:10.1126/science.aan8630 28931639 PMC5978417

[41] Wilkinson I, Anderson S, Fry J, Julien LA, Neville D, Qureshi O, Watts G, Hale G. 2021. Fc-engineered antibodies with immune effector functions completely abolished. PLoS One 16:e0260954. doi:10.1371/journal.pone.0260954 34932587 PMC8691596

[42] Martínez-Sobrido L, García-Sastre A. 2010. Generation of recombinant influenza virus from plasmid DNA. J Vis Exp 2010:42. doi:10.3791/2057 PMC3156010 20729804

[43] Margine I, Palese P, Krammer F. 2013. Expression of functional recombinant hemagglutinin and neuraminidase proteins from the novel H7N9 influenza virus using the Baculovirus expression system. J Vis Exp 2013:e51112. doi:10.3791/51112 PMC3970794 24300384

[44] Roubidoux EK, Carreño JM, McMahon M, Jiang K, van Bakel H, Wilson P, Krammer F. 2021. Mutations in the hemagglutinin stalk domain do not permit escape from a protective, stalk-based vaccine-induced immune response in the mouse model. mBio 12:e03617-20. doi:10.1128/mBio.03617-20 33593972 PMC8545130

[45] Mazor Y, Barnea I, Keydar I, Benhar I. 2007. Antibody Internalization studied using a novel IgG binding toxin fusion. J Immunol Methods 321:41–59. doi:10.1016/j.jim.2007.01.008 17336321

