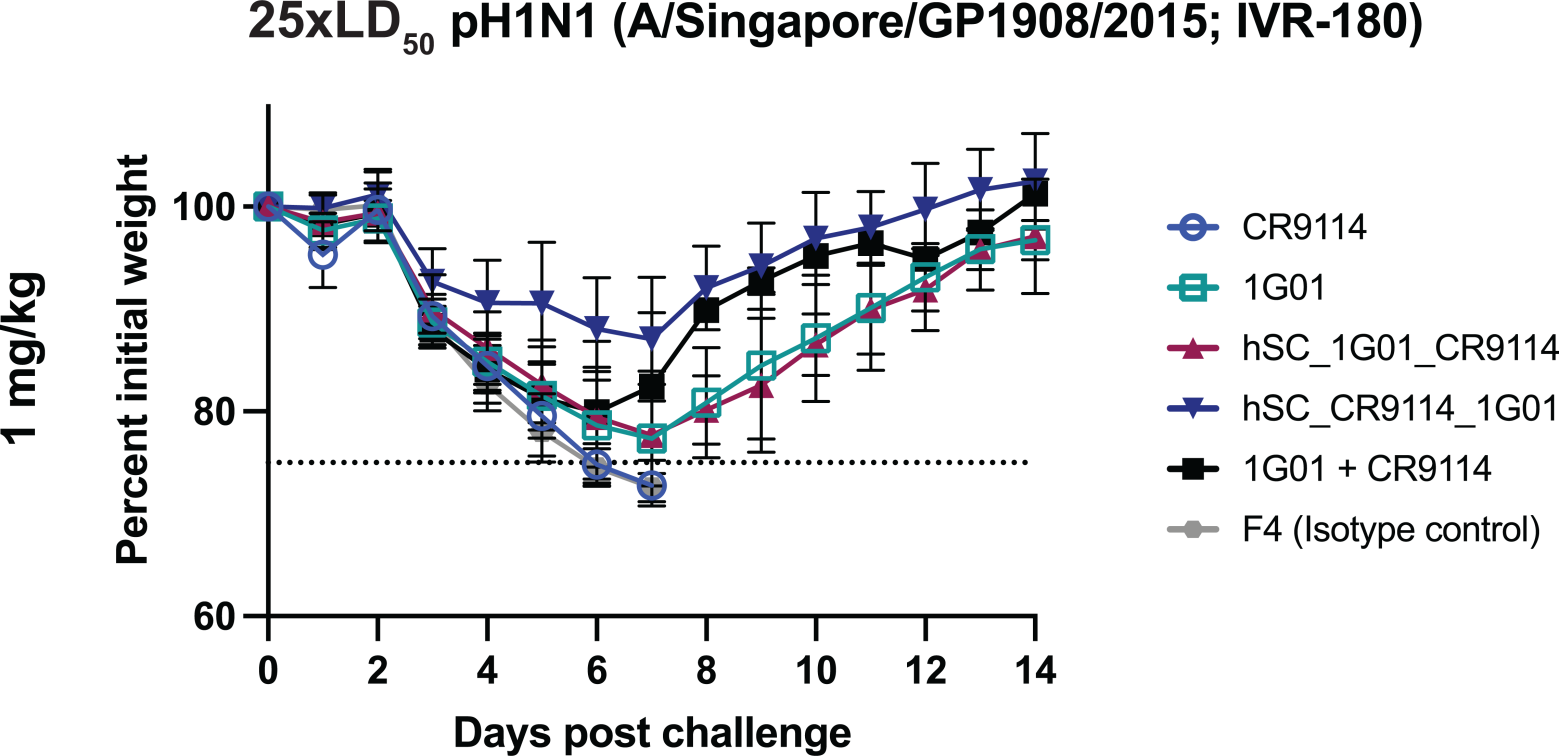# Correction for Ramos et al., “Broadly protective bispecific antibodies that simultaneously target influenza virus hemagglutinin and neuraminidase”

**DOI:** 10.1128/mbio.00278-25

**Published:** 2025-03-04

**Authors:** Kevin E. Ramos, Nisreen M. A. Okba, Jessica Tan, Pooja Bandawane, Philip Meade, Madhumathi Loganathan, Benjamin Francis, Sergey Shulenin, Frederick W. Holtsberg, M. Javad Aman, Meagan McMahon, Florian Krammer, Jonathan R. Lai

## AUTHOR CORRECTION

Volume 15, no. 7, e01085-24, 2024, https://doi.org/10.1128/mbio.01085-24. Page 9, Fig. 5: Panel 3 in row 2 should appear as shown in this correction. The data for the 1G01 + CR9114 cocktail group were inadvertently plotted for the 5 mg/kg dose (duplicated from the panel above) instead of the 1 mg/kg dose. The text describes the correct data, and thus there are no changes to the conclusions. We apologize for any confusion this may have caused.

**Fig 5:**