# Supplementary material for: Broadly protective bispecific antibodies that simultaneously target influenza virus hemagglutinin and neuraminidase
Source: mBio. 2024 Jun 20;15(7):e01085-24. doi: 10.1128/mbio.01085-24 (PMC11253627; doi:10.1128/mbio.01085-24)
Supplement: Supplemental material — Supplemental figures and tables. [file mbio.01085-24-s0001.docx]

**Supporting Information for: Broadly protective bispecific antibodies that simultaneously target influenza hemagglutinin and neuraminidase**


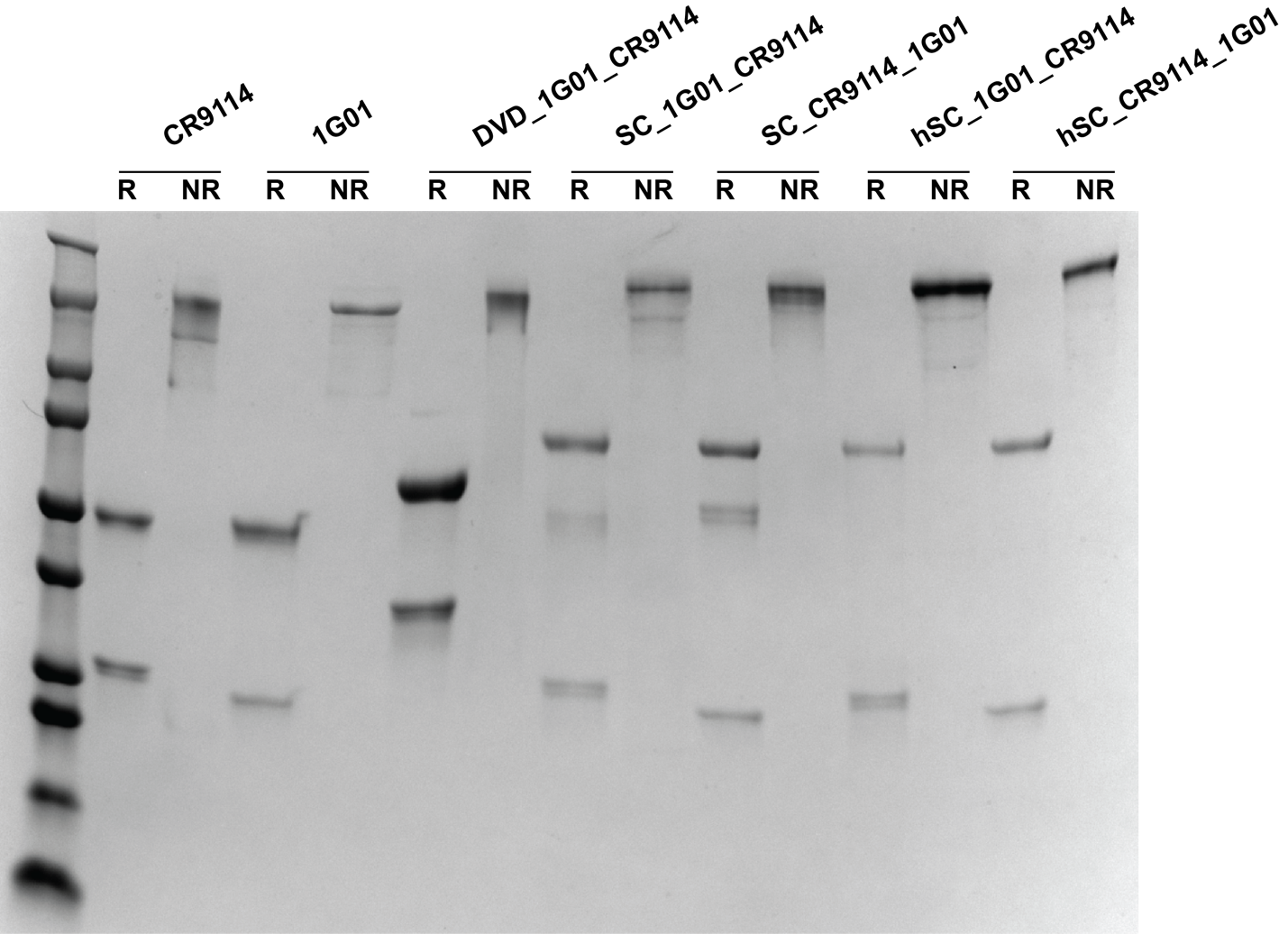


**Figure S1.** Parental mAbs and bsAbs were analyzed by a SDS-polyacrylamide gel under reducing (R) and nonreducing (NR) conditions, which were stained with Coomassie Brilliant Blue to visualize.


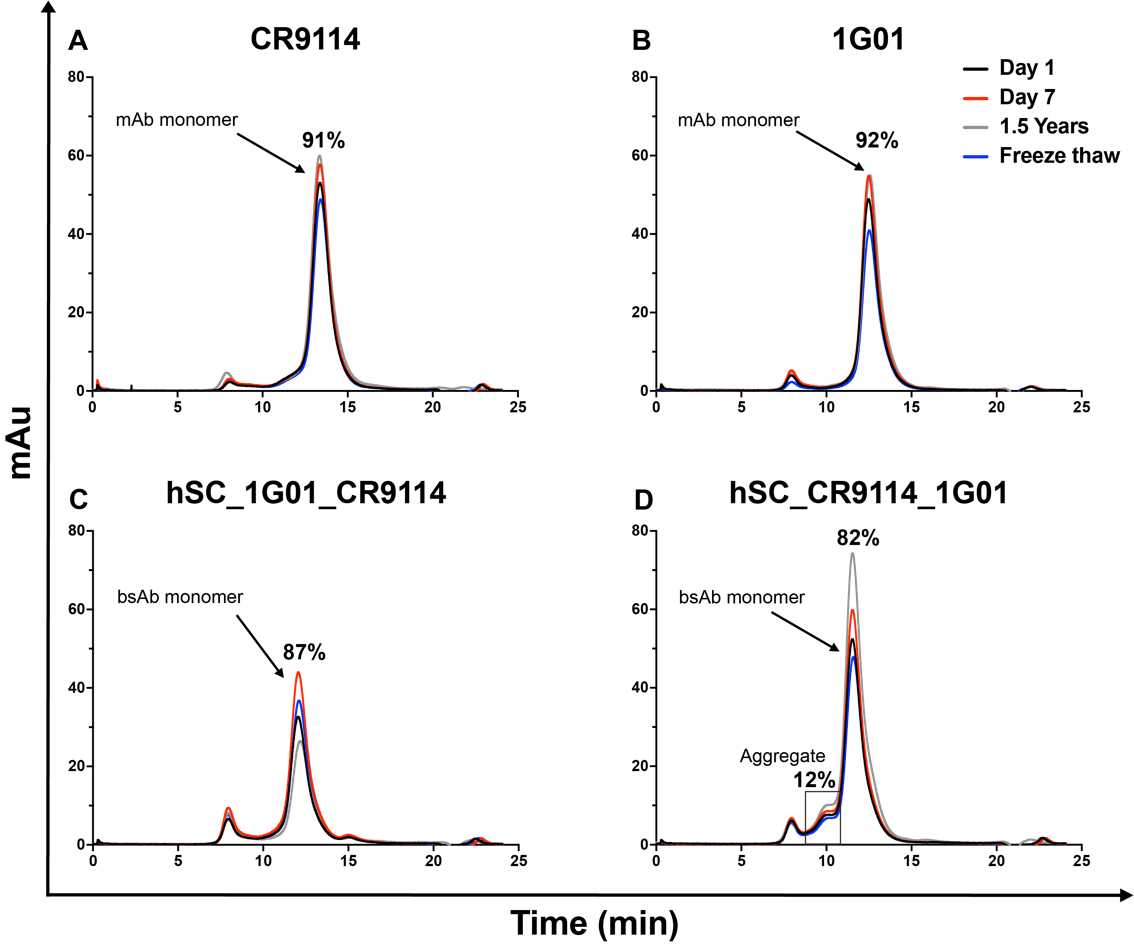


**Figure S2.** Parental mAbs, CR9114 and 1G01 (A-B) and bsAbs, hSC_1G01_CR9114 and hSC_CR9114_1G01 (C-D) were analyzed by size-exclusion chromatography to further assess any aggregation using a Superdex 200 Increase 10/300 GL column that was equilibrated with 150 mM HEPES and 200 mM NaCl at pH 7.4.


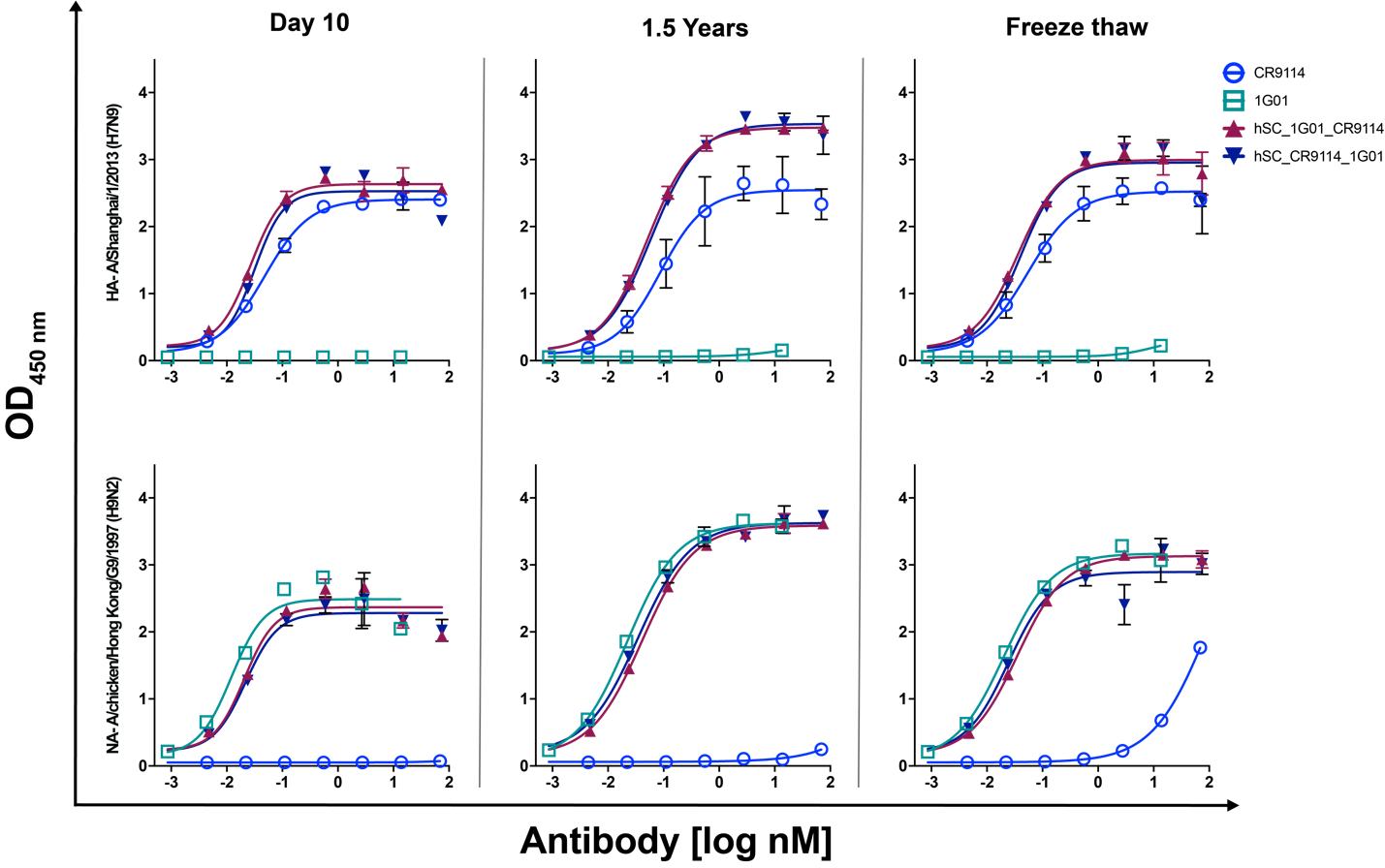


**Figure S3.** ELISA curves of Day 10, 1.5 Years and freeze thaw samples of mAbs and bsAbs against HA and NA antigens.

|  | | | |
| --- | --- | --- | --- |
|  |  | EC_50_ (nM) | |
|  | Antibody | HA | NA |
| Day 10 | CR9114 | 0.05 | N.D. |
|  | 1G01 | N.D. | 0.01 |
|  | hSC_1G01_CR9114 | 0.03 | 0.02 |
|  | hSC_CR9114_1G01 | 0.03 | 0.02 |
| 1.5 Years | CR9114 | 0.09 | N.D. |
|  | 1G01 | N.D. | 0.02 |
|  | hSC_1G01_CR9114 | 0.05 | 0.04 |
|  | hSC_CR9114_1G01 | 0.06 | 0.03 |
| Freeze thaw | CR9114 | 0.05 | N.D. |
|  | 1G01 | N.D. | 0.02 |
|  | hSC_1G01_CR9114 | 0.04 | 0.04 |
|  | hSC_CR9114_1G01 | 0.04 | 0.02 |

**Table S1.** Summary of EC_50_ values (in nM) derived from curve fitting of Day 10, 1.5 Years and freeze thaw samples of mAbs and bsAbs against HA and NA antigens.


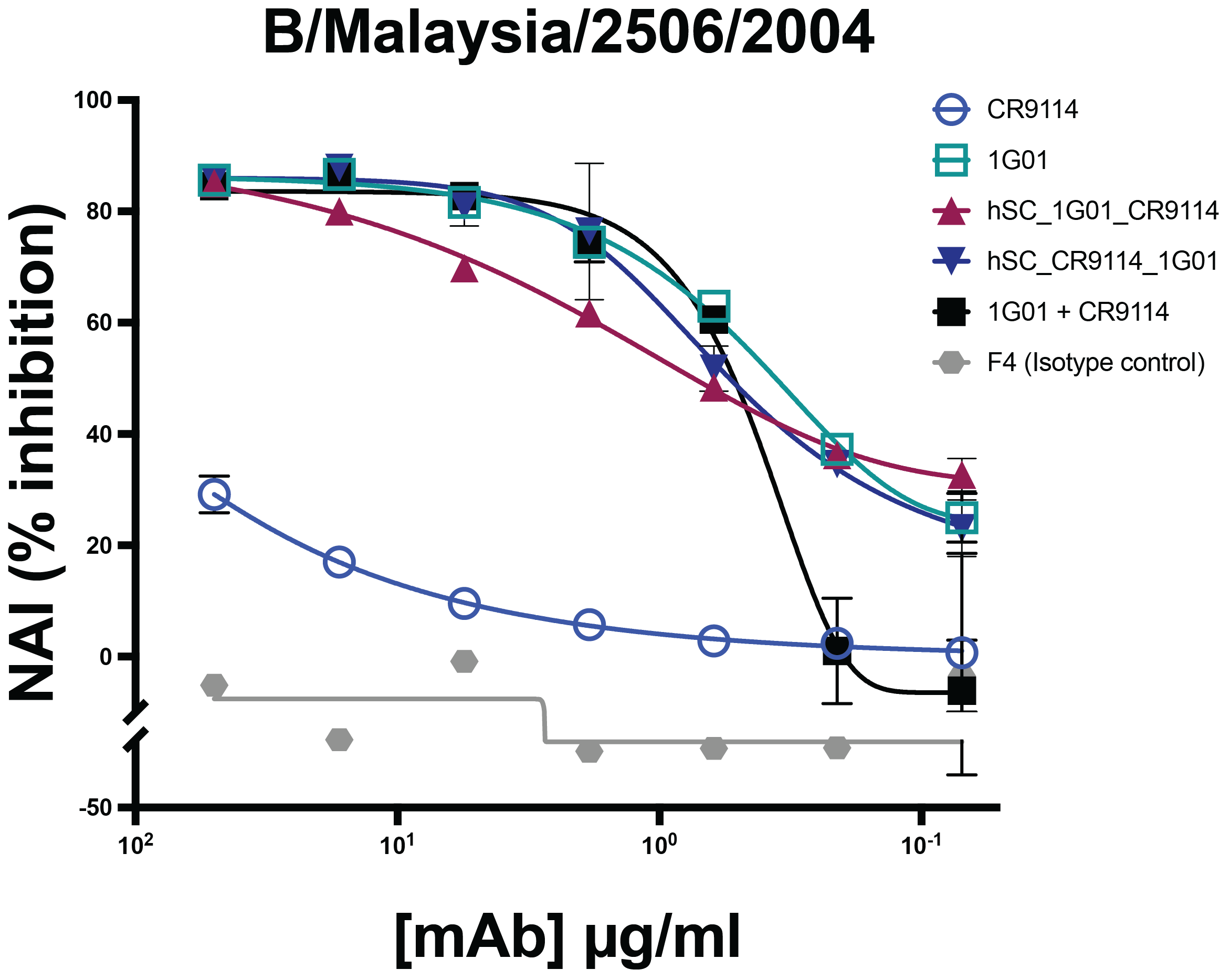


|  | CR9114 | 1G01 | hSC_1G01_CR9114 | hSC_CR9114_1G01 | 1G01 + CR9114 | F4 (Isotype control) |
| --- | --- | --- | --- | --- | --- | --- |
| EC_50_ | 25.67 | 0.47 | 1.97 | 0.55 | 0.43 | Unstable |

**Figure S4**. Neuraminidase inhibitory (NAI) activity of the bsAbs. The ability of the parental mAbs (individually and as a cocktail) and bsAbs to inhibit neuroaminidase (NA) activity was determined with an enzyme-linked lectin assay **(**ELLA). The table summarizes the 50% effective concentration (EC_50_) values of each antibody tested.


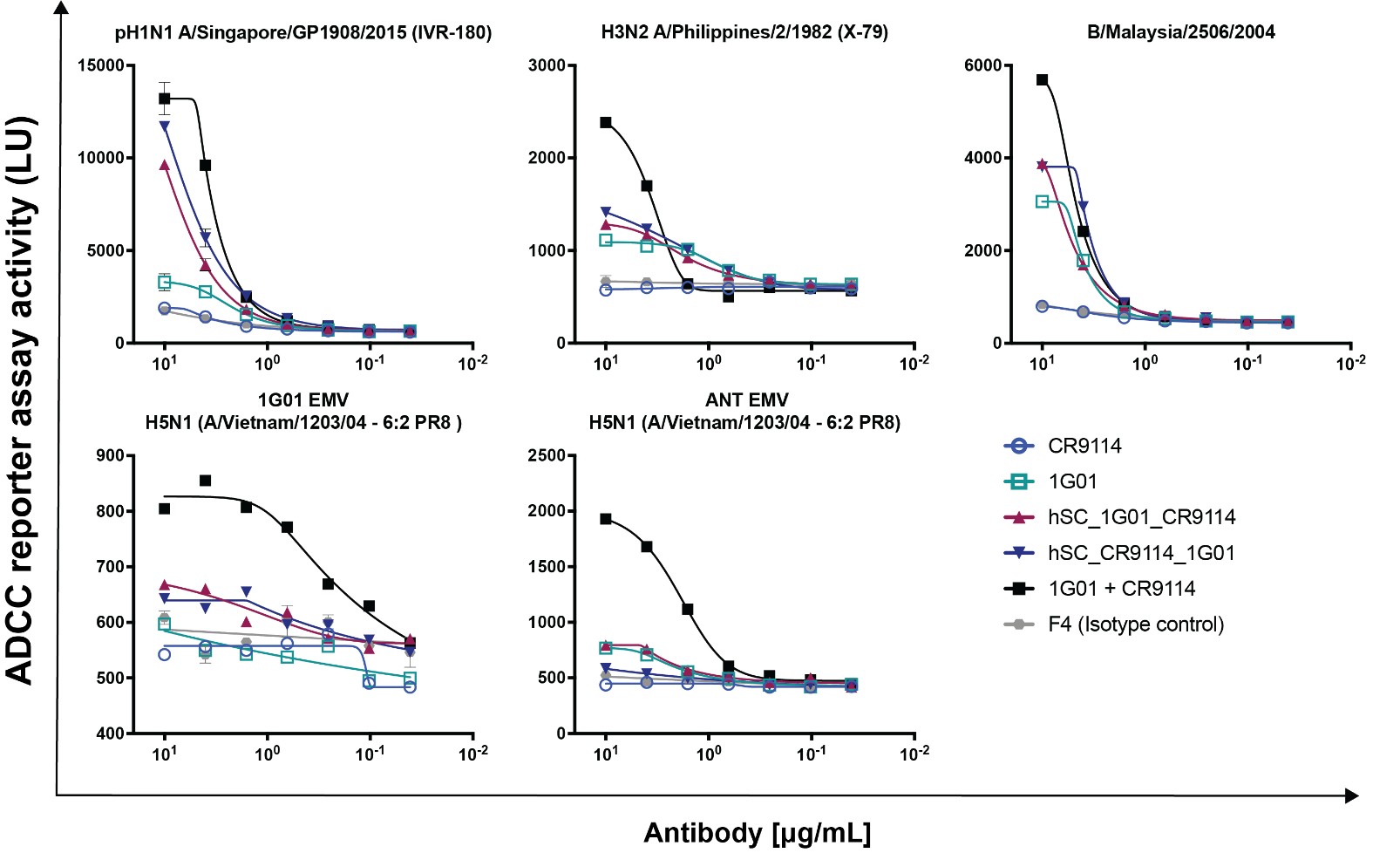


**Figure S5**. Antibody dependent cellular cytotoxicity (ADCC) activity of the bsAbs. The ADCC activity of the parental mAbs (individually and as a cocktail) and bsAbs against various viruses was measured using a luciferase reporter assay. LU, luminescence units.


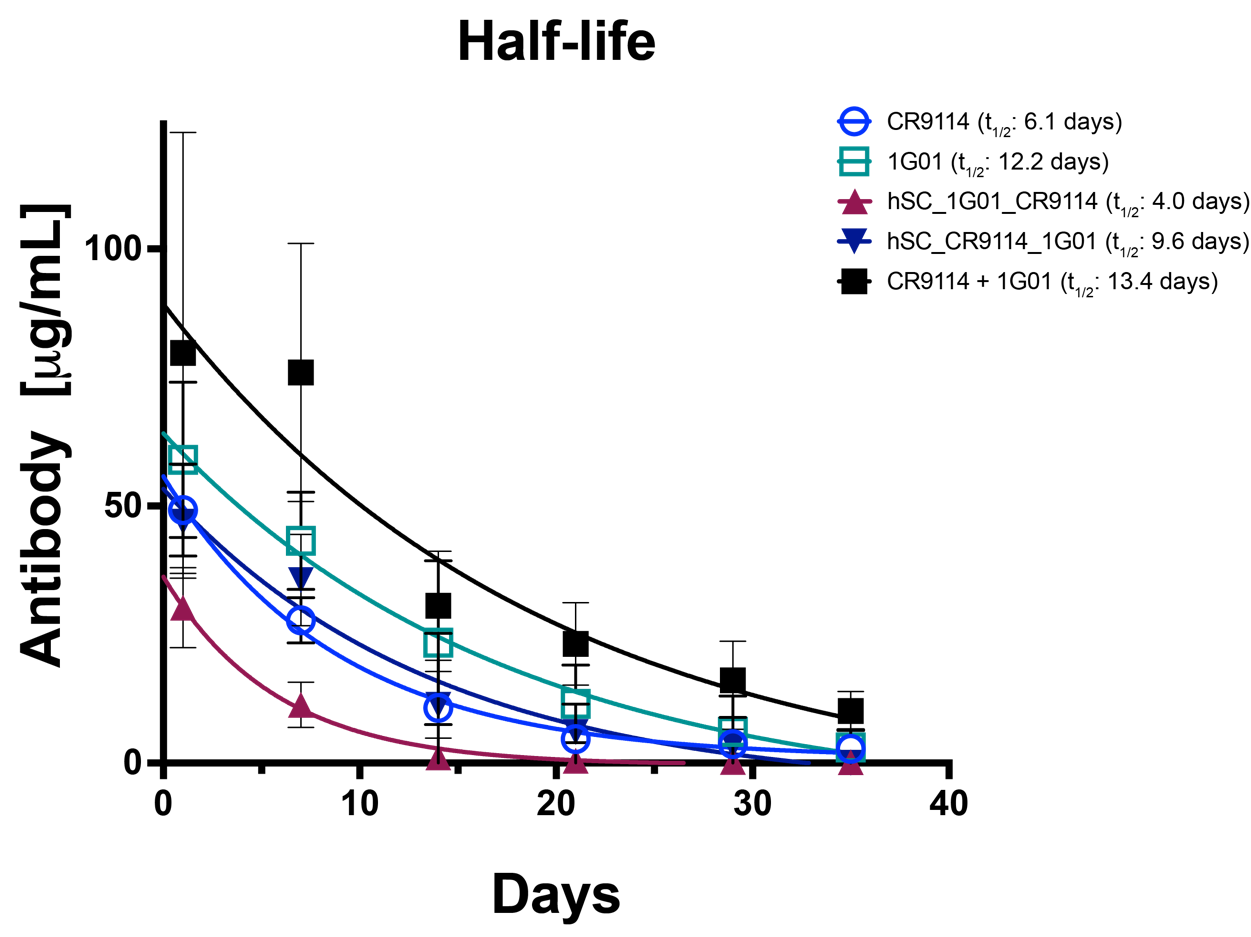


**Figure S6**. Half-life of antibodies was monitored in vivo for 35 days. The half-life for each antibody is indicated in parenthesis next to the legend. The dose of all mAbs and bsAbs was 5 mg/kgs; for the CR9114 + 1G01 cocktail, the dose was 5 mg/kgs for each mAb.

**
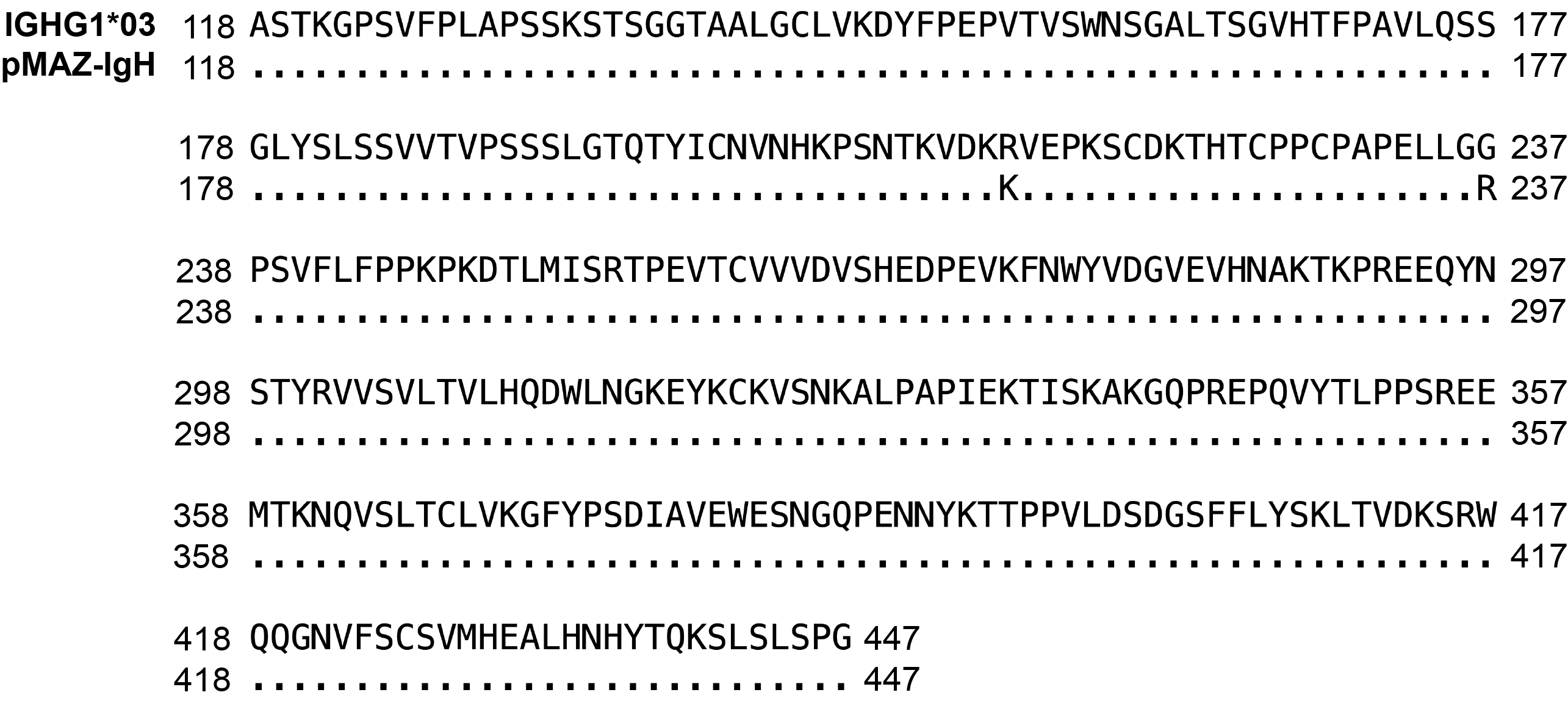
**

**Figure S7**. Sequence alignment of Fc regions from pMAZ-IgH expression vector used in this study, and common IgG1 allotype IGHG1*03. The mutations on pMAZ-IgH are indicated (R214K and G237R), which are presumed to decrease ADCC activity.

## Table S2. Virus strains used in this study

| **Virus** | **Subtype** | **HA** | **NA** | **Backbone** |
| --- | --- | --- | --- | --- |
| A/Singapore/GP1908/2015 (IVR-180; pH1N1) | pH1N1 | A/Singapore/GP1908/2015 | A/Singapore/GP1908/2015 | A/Texas/1/1977 |
| A/Vietnam/1203/2004 - 6:2 PR8 | H5N1 | A/Vietnam/1203/2004  (polybasic cleavage site removed) | A/Vietnam/1203/2004 | A/Puerto Rico/8/1934 |
| A/Shanghai/1/2013 - 6:2 PR8 | H7N9 | A/Shanghai/1/2013 | A/Shanghai/1/2013 | A/Puerto Rico/8/1934 |
| A/Philippines/2/1982 (X-79) | H3N2 | A/Philippines/2/1982 | A/Philippines/2/1982 | A/Puerto Rico/8/1934 |
| A/mallard/Interior Alaska/10BM01929/2010 | H10N7 | A/mallard/Interior Alaska/10BM01929/2010 | A/mallard/Interior Alaska/10BM01929/2010 | wild type |
| A/Netherlands/602/2009 | pH1N1 | A/Netherlands/602/2009 | A/Netherlands/602/2009 | wild type |
| A/Netherlands/602/2009 CR9114 EMV ((PMID: 33593972) | pH1N1 | A/Netherlands/602/2009 | A/Netherlands/602/2009 | wild type |
| A/Vietnam/03/2004 - 6:2 PR8 1G01 EMV | H5N1 | A/Vietnam/1203/2004 (polybasic cleavage site removed) | A/Vietnam/1203/2004 | A/Puerto Rico/8/1934 |
| A/mallard/Sweden/86/2003 | H12N5 | A/mallard/Sweden/86/2003 | A/mallard/Sweden/86/2003 | wild type |
| B/Malaysia/2506/2004 | B/Victoria/2/1987-like | B/Malaysia/2506/2004 | B/Malaysia/2506/2004 | wild type |
| B/Florida/04/2006 | B/Yamagata/16/1988-like | B/Florida/04/2006 | B/Florida/04/2006 | wild type |

## Table S3. Recombinant proteins used in this study

| **Protein subtype** | **Protein name** | **Strain name (subtype)** |
| --- | --- | --- |
| H1 | H1 prepdm | A/New Caledonia/20/1999(H1N1) |
| H1 | H1 Mich/15 | A/Michigan/45/2015 (H1N1) |
| H2 | H2 | A/Ann Arbor/6/1960 (H2N2) |
| H5 | H5 | A/Vietnam/1203/2004 (H5N1) |
| H6 | H6 | A/Taiwan/2/2013 (H6N1) |
| H9 | H9 | A/chicken/Hong Kong/G9/1997 (H9N2) |
| H12 | H12 | A/mallard/Interior Alaska/7MP0167/2007(H12N5) |
| H8 | H8 | A/mallard/Sweden/24/2002 (H8N4) |
| H11 | H11 | A/shoveler/Netherlands/18/1999 (H11N9) |
| H13 | H13 | A/black headed gull/Sweden/1/1999 (H13N6) |
| H16 | H16 | A/black headed gull/Sweden/5/1999 (H16N3) |
| H17 | H17 | A/yellow shouldered bat/Guatemala/06/2010 (H17N10) |
| H18 | H18 | A/bat/Peru/33/2010 (H18N11) |
| H3 | H3 | A/Indiana/10/2011 (H3N2) |
| H3 | H3 Phil/82 | A/Philippines/2/1982 (H3N2) |
| H3 | H3 HK/14 | A/Hong Kong /4801/2014 (H3N2) |
| H3 | H3 HK/68 | A/Hong Kong/1/1968 (H3N2) |
| H3 | H3 Kansas/17 | A/Kansas/14/2017 (H3N2) |
| H4 | H4 | A/red knot/Delaware/541/1988 (H4N6) |
| H14 | H14 | A/mallard/Gurjev/263/1982 (H14N5) |
| H7 | H7 | A/Shanghai/1/2013 (H7N9) |
| H10 | H10 | A/Jiangxi-Donghu/346/2013 (H10N8) |
| H15 | H15 | A/shearwater/West Australia/2576/1979 (H15N9) |
| B (Y) HA | B/Phuk | B/Phuket/3073/2013 (B/Yamagata/16/1988-like) |
| B (V) HA | B/Wash 1&2 | B/Washington/02/2019 (B/Victoria/2/1987-like) |
| B (V) HA | B/mal HA | B/Malaysia/2506/2004 (B/Victoria/2/1987-like) |
| B (V) NA | B/mal NA | B/Malaysia/2506/2004 (B/Victoria/2/1987-like) |
| B (Y) NA | B/Yam NA | B/Yamagata/16/1988 |
| B (Y) HA | B/Yam HA | B/Yamagata/16/1988 |
| N2 | N2 | A/chicken/Hong Kong/G9/1997 (H9N2) |
